# Integrated analysis of WGCNA and machine learning identified diagnostic biomarkers in dilated cardiomyopathy with heart failure

**DOI:** 10.3389/fcell.2022.1089915

**Published:** 2022-12-05

**Authors:** Yihao Zhu, Xiaojing Yang, Yao Zu

**Affiliations:** ^1^ International Research Center for Marine Biosciences, Ministry of Science and Technology, Shanghai Ocean University, Shanghai, China; ^2^ Key Laboratory of Exploration and Utilization of Aquatic Genetic Resources, Ministry of Education, Shanghai Ocean University, Shanghai, China; ^3^ Marine Biomedical Science and Technology Innovation Platform of Lin-gang Special Area, Shanghai, China

**Keywords:** dilated cardiomyopathy, heart failure, diagnostic biomarker, weighted gene coexpression network analysis, machine learning

## Abstract

The etiologies and pathogenesis of dilated cardiomyopathy (DCM) with heart failure (HF) remain to be defined. Thus, exploring specific diagnosis biomarkers and mechanisms is urgently needed to improve this situation. In this study, three gene expression profiling datasets (GSE29819, GSE21610, GSE17800) and one single-cell RNA sequencing dataset (GSE95140) were obtained from the Gene Expression Omnibus (GEO) database. GSE29819 and GSE21610 were combined into the training group, while GSE17800 was the test group. We used the weighted gene co-expression network analysis (WGCNA) and identified fifteen driver genes highly associated with DCM with HF in the module. We performed the least absolute shrinkage and selection operator (LASSO) on the driver genes and then constructed five machine learning classifiers (random forest, gradient boosting machine, neural network, eXtreme gradient boosting, and support vector machine). Random forest was the best-performing classifier established on five Lasso-selected genes, which was utilized to select out NPPA, OMD, and PRELP for diagnosing DCM with HF. Moreover, we observed the up-regulation mRNA levels and robust diagnostic accuracies of NPPA, OMD, and PRELP in the training group and test group. Single-cell RNA-seq analysis further demonstrated their stable up-regulation expression patterns in various cardiomyocytes of DCM patients. Besides, through gene set enrichment analysis (GSEA), we found TGF-β signaling pathway, correlated with NPPA, OMD, and PRELP, was the underlying mechanism of DCM with HF. Overall, our study revealed NPPA, OMD, and PRELP serving as diagnostic biomarkers for DCM with HF, deepening the understanding of its pathogenesis.

## Introduction

As the most common primary cardiomyopathy, dilated cardiomyopathy (DCM), is characterized by left ventricular dilation along with systolic dysfunction (Weintraub et al., 2017). The clinical manifestation of DCM ranges from none to overt symptoms of heart failure (HF) (McNally and Mestroni, 2017). Accordingly, there is no specific clinical manifestation for the diagnosis of DCM. Most patients with DCM usually have a progressively worsening condition, leading to advanced HF and ultimate death (Mahmaljy et al., 2022; Zheng et al., 2022). DCM is an essential cause of HF, and advanced HF in DCM is a leading indication for heart transplantation (Seferović et al., 2019). The estimated prevalence of DCM is 1 case per 250 individuals (Seferović et al., 2019). Notably, the reported incidence of DCM in patients with HF varied greatly (8%–47%) due to the precise diagnosis lacking (Seferović et al., 2019). There are many etiologies of DCM, most of which are idiopathic (McNally and Mestroni, 2017; Mahmaljy et al., 2022; McDonagh et al., 2022). Etiologies of HF in DCM mainly include inherited (pathogenic gene mutations) and various acquired causes (infectious or toxic agents, auto-immunity, endocrine or metabolic abnormalities) (Seferović et al., 2019; McDonagh et al., 2022). To date, mutations in genes coding for cytoskeletal, contractile proteins, nuclear membrane, ion channels, and intercellular junction molecules has been proven to be associated with DCM (Japp et al., 2016; Seferović et al., 2019). Among these genetic variants in DCM, inherited mutations of TTN, LMNA, MYH7, MYH6, TNNT2, and ACTC1 are the leading causes (Fatkin et al., 1999; Seidman and Seidman, 2001; Gerull et al., 2002; Carniel et al., 2005; Mazzarotto et al., 2020). During recent decades, continuously improving diagnosis and treatment strategies (including early diagnosis development, advanced pharmacological treatments, and device therapies) have greatly improved the prognosis and survival of DCM patients (Merlo et al., 2014). However, the etiologies and pathogenesis of DCM with HF have not been fully understood, resulting in non-specific treatment. Heart transplantation remains the only viable approach for treating DCM with advanced HF (Iwata et al., 2020). Therefore, novel mechanisms needed to be elucidated to improve this situation. Besides, developing a precise diagnosis approach for DCM with HF is equally significant.

In this study, we conducted a series of bioinformatics analyses and machine learning classifiers to identify the underlying genes as diagnostic biomarkers for DCM with HF patients, which might provide insight into diagnosis and treatment for DCM with HF. The overall study design was shown in Supplementary Figure S1. First, three gene expression profiling datasets of DCM with HF (GSE29819, GSE21610, GSE17800) were downloaded from the GEO database. GSE29819 and GSE21610 were merged into the training group, while GSE17800 was the test group for validation. Second, we performed Principal Component Analysis (PCA) on the training group to evaluate data dispersion and then screened out the differential expression genes (DEGs) between the control and DCM with HF patients. Third, we explored the potential biological functions, signaling pathways, and correlated diseases of the up-regulated DEGs. Fourth, the gene co-expression network was constructed, and driver genes of identified module closely associated with DCM with HF were extracted. Next, we identified the intersection of the driver genes and up-regulated genes as the feature genes for DCM with HF. Fifth, within the training and test groups, Back Propagation Neural Network (BPNN) established on these feature genes was utilized to classify the normal and DCM with HF patients (Ruan et al., 2021). We assessed the classified accuracy of BPNN with the Area Under Curve (AUC) score of the Receiver Operating Characteristic (ROC) curve. Sixth, the least absolute shrinkage and selection operator (Lasso) was first conducted to narrow down the feature gene sets and five well-established classifiers (Random Forest, Gradient Boosting Machine, Neural Network, eXtreme Gradient Boosting, Support Vector Machine) (Han et al., 2014; Sheridan et al., 2016; Olson et al., 2018; Yang et al., 2020; Ruan et al., 2021) on the Lasso-selected features were applied. In the training group and test group, the classifier performed with the minimum residual and maximum AUC score was considered the most robust for selecting the critical features (key genes) as biomarkers for diagnosing DCM with HF (Pan et al., 2021). Seventh, we analyzed the mRNA expression levels and diagnosis abilities of key genes in the training group and test group. Moreover, we carried out single-cell RNA-sequencing analysis on GSE95140, thus demonstrating the expression distributions and dynamic changes of key genes in damaged cardiomyocytes of DCM patients. We then investigated and inferred the potential pathways of key genes by integrating the Gene Set Enrichment Analysis (GSEA) with Pearson’s correlation analysis. Overall, our research identified promising diagnostic biomarkers from integrating WGCNA and machine learning and might be a fresh perspective to understand the development of DCM with HF.

## Materials and methods

### Data collection and processing

Three gene expression profiles of DCM with HF (GSE29819, GSE21610, GSE17800) and one single-cell RNA-sequencing dataset of DCM (GSE95140) were downloaded from the GEO database (https://www.ncbi.nlm.nih.gov/geo/). First, GSE29819 and GSE21610 were merged into the training group for major analysis, containing 20 normal myocardial tissues and 35 myocardial tissues derived from DCM patients with HF (Schwientek et al., 2010; Gaertner et al., 2012). GSE17800 was the test group for validation, including nine normal myocardial tissues and 40 myocardial tissues from DCM patients with HF (Ameling et al., 2013). GSE29819, GSE21610, and GSE17800 were based on the GPL570 sequencing platform. And a total of 21,628 genes and 21,654 genes were obtained in the training group and test group, respectively. The probes were annotated to official gene symbols using the Perl programming. Next, we performed log2 transformation and then normalized the raw count expression data using the function “normalizeBetweenArrays” of the R package “limma”. For the training group, the function “ComBat” of the R package “sva” was then utilized to remove the batch effect of GSE29819 and GSE21610. Moreover, heart specimens from normal individuals and 10 DCM patients were obtained from GSE95140 for further verification and single-cell RNA-seq analysis (Nomura et al., 2018). The sequencing platform of GSE95140 was GPL16791.

### Principal component analysis and identification of DEGs

To assess the dispersion between the control group and DCM with HF group, PCA was performed and two components were extracted. Then, the R package “limma” was utilized to screen the DEGs between control and DCM with HF samples. The cutoff criteria of DEGs was |log_2_Fold Change (log_2_FC) | > 1 and adjusted *p* < 0.05 (Chen et al., 2018). The Volcano plot of all DEGs and the clustering heatmap of the top 50 up-regulated genes and top 50 down-regulated genes were plotted by the R packages “ggplot2” and “pheatmap”, respectively.

### Gene functional, pathway, and, disease enrichment analysis of up-regulated DEGs

To investigate the potential biological function, pathways, and related diseases of up-regulated DEGs, the R packages “clusterProfiler” and “DOSE” were used for Gene Ontology (GO), Kyoto Encyclopedia of Genes and Genomes (KEGG), and Disease Ontology (DO) enrichment analyses. The enrichment GO terms, KEGG pathways, and DO terms with *q* < 0.05 were considered significant. Furthermore, we used the R package “CBNplot” to infer the Bayesian network (BN) on the gene expression profiles and KEGG enrichment analysis, showing the relationships between genes in the enriched pathways (Sato et al., 2022).

### Construction of gene Co-expression network and identification of the modules

The R package “WGCNA” was used to construct the gene co-expression network and identify the modules correlated to DCM with HF (Langfelder and Horvath, 2008). First, a total of 9,379 genes with standard deviation (SD) of expression larger than 0 in the training group were selected for analysis. Second, the function “hclust” was used to cluster the samples to exclude the outliers. Third, a similarity matrix was obtained by calculating each pairwise gene correlation. It was then converted to an adjacency matrix using the soft adjacency function $A_{ij}={\left|S_{ij}\right|}^{\beta }$ , where *i* and *j* were any two of 9,379 genes, *β* is the soft threshold power, *S*_*ij*_ is the similarity matrix, and *A*_*ij*_ is the adjacency matrix. The function “pickSoftThreshold” selected the optimal soft threshold power (*β* = 1–20) to establish the scale-free topology. The optimal soft threshold power was also graphically validated by the high scale-free topology *R* ^2^ between log10(k) and log10 (p(k)), where k is the connectivity between genes and p(k) means the probability of connectivity. Fourth, the adjacency matrix was turned into a Topological Overlap Matrix (TOM). The functions “pickSoftThreshold” and “plotDendroAndColors” were conducted to identify the modules and then plot the clustering dendrogram of genes together with dissimilarity on the topological overlap and various module colors. Herein, the parameters “minModuleSize” and “MEDissThres” were set to 60 and 0.3, respectively. Fifth, module-trait correlation analysis was performed to reveal the ME blue module as the most important one related to DCM with HF. Subsequently, the Gene significance (GS) and Module Membership (MM) were calculated by intramodular analysis. Genes with GS > 0.5 and MM > 0.8 were identified as driver genes in the ME blue module for further study (Ai et al., 2020).

### Construction and validation of BPNN on feature genes

The intersection of up-regulated DEGs and driver genes in the ME blue module was identified as feature genes for DCM with HF. Next, the clustering heatmap of the feature gene expression was plotted using the R package “pheatmap”. BPNN, typically composed of processing elements arranged in at least three layers (input, hidden, and output layers), is a multilayer feedforward network trained on an error-back propagation algorithm (Kaundal et al., 2006). Here, BPNN was constructed in the training group based on feature genes and then validated in the test group using the R package “neuralnet”. For BPNN, the input and output layer neurons are equal to the feature gene number and the classification number (control or DCM with HF), respectively. According to common rule-of-thumb methods, the hidden layer neurons were determined and adjusted by 1/3–2/3 of the input layer neurons (Karsoliya, 2012). Accordingly, the neuron numbers of the input layer, hidden layer, and output layer were set as 13, 5, and 2, respectively. The classification accuracies of BNPP in the training and test groups were evaluated by the AUC score of the ROC curve by the R package “pROC”. Additionally, we visualized Pearson’s correlation among the 13 feature genes by the R package “corrplot”.

### Construction and evaluation of RF, GBM, NN, XGBoost, and SVM

To narrow down the numbers of feature genes, Lasso was first performed to carry out feature selection. Five machine learning classifiers (RF, GBM, NN, XGBoost, and SVM) were then constructed for further feature selection on the Lasso-selected genes (Han et al., 2014). These models were all constructed through 10-fold cross-validation. The setting paraments of RF, NN, and SVM were 400 decision trees, five neurons in the hidden layer, and radial kernel. And paraments set by default for GBM and XGBoost. By using the R package “glmnet”, Lasso was first applied to reduce gene numbers for screening the feature genes. Then, five machine learning classifiers were established in the training and test groups based on the Lasso-selected feature genes using the R package “caret”. The classification performances of five models in the training and test groups were assessed by AUC scores using the package “pROC”. The residual and feature importance of classifiers were analyzed by the R package “DALEX”. Net Benefit (NB) calculated by Decision Curve Analysis (DCA) and Brier score were also utilized to measure the clinical value and calibration of classifiers, respectively (Fitzgerald et al., 2015; Alba et al., 2017). In general, the classifier with the lowest residual, minimum Brier score, highest AUC, and maximum NB was considered the best predictive classifier for further feature selection. Herein, RF was selected as the best-performing classifier for accurately distinguishing between normal and DCM with HF groups. Using the LASSO-selected genes, the R packages “caret” and “randomForest” were used to build an RF classifier in the training and test groups. The feature importance of RF was investigated by using the Gini gain approach. Feature genes with a mean decrease Gini larger than two were selected as key genes for diagnosing DCM with HF (Tian et al., 2020).

### Differentially expression, diagnostic accuracy, and target therapeutic drug prediction analyses of key genes

Then, within the training and test groups, we analyzed the expression distribution and diagnostic accuracy of key genes selected by RF. By using the R package “ggpubr”, the boxplots were plotted and demonstrated the differential expression distributions of key genes between control and DCM with HF groups. The diagnostic accuracies of key genes for DCM with HF were evaluated by the AUC score of the ROC curve using the R packages “pROC”. Furthermore, we established a logistic regression model for evaluating the diagnostic accuracy of the key genes’ combination using the R package “glmnet”. Furthermore, the nomogram was used to illustrate and visualize the logistic regression model by the R package “rms”. The calibration curve and NB calculated by DCA were utilized to evaluate the prediction effect of the nomogram. The gene with an AUC score ranging from 0.85 to 1.0 was considered to have excellent specificity and sensitivity to distinguish between the control group and DCM with HF group. The Human Protein Atlas (HPA) database (https://www.proteinatlas.org/) was used to explore the extracellular locations and tissue-specific expression levels of the key genes. Moreover, Drug Signatures Database (DSigDB) (https://maayanlab.cloud/Enrichr/) (Kuleshov et al., 2016) was applied to predict potential therapeutic drugs target for three key genes, with adjusted *p* < 0.05 as the filtering threshold.

### Assessment of key genes expression on single-cell RNA-seq data

To further confirm the differential expression of key genes in DCM with HF, we performed single-cell RNA-seq analysis on GSE95140, mainly including cell type identification and Pseudotime-ordered analysis. First, we performed quality control and data cleaning using the R package “dplyr”. The screening criteria were set: the number of genes in each cell ranges from 200 to 7,500, and the percentage of mitochondrial genes in each cell is lower than 20%. Second, the function “NormalizeData” of the R package “seruat” was used to perform log2 transformation on the raw count data. Next, the top 2000 hypervariable genes were selected by the function “FindVariableFeatures”. Z-score standardization was conducted using the function “ScaleData”. Third, PCA was carried out on the hypervariable genes in the control and DCM groups. Next, two PCs (control group, all *p* < 0.01) and four PCs (DCM group, all *p* < 0.01) were selected. Then, two clusters (control group) and four clusters (DCM group) were obtained and projected onto UMAP plots, respectively. Fourth, the marker genes of cardiomyocyte (NNT2, MYH7), fibroblast (DCN, COL1A1), and endothelial cell (CDH5, VWF) obtained from CellMarker 2.0 database (http://yikedaxue.slwshop.cn/) (Zhang et al., 2019) were used to identify cardiomyocytes in different states. We then explored the expression distribution of key genes and NPPB in the control and DCM groups. NPPB, an important paralog of NPPA with up-regulation in DCM with HF (Barth et al., 2006), was selected as an indicator to reveal whether the key genes showed co-expression and dynamic expression patterns of NPPB. Fifth, the R package “monocle” was used to conduct Pseudotime-ordered analysis using the “DDRTree” method on the DCM group. Using the function “plot_cell_trajectory”, the differentiation trajectory of cardiomyocytes was visualized in the tree diagram. Additionally, we analyzed and visualized the dynamic expression patterns of key genes and NPPB during the cardiomyocyte transitions across the Pseudotime using the function “plot_genes_in_pseudotime”.

### Inference of pathways regulated by key genes in DCM with HF

Considering the limitation of performing KEGG on small gene sets, GSEA was utilized to explore the potential pathways of key genes. We conducted GSEA on the training group using the R package “clusterProfiler”. The KEGG gene sets annotated for GSEA (c2.cp.kegg.v7.5.1.symbols) were downloaded from the MSigDB database (http://www.gsea-msigdb.org/gsea/msigdb/index.jsp). First, the samples were divided into low- and high-expression groups according to the median expression of NPPA, OMD, and PRELP, respectively. Then, we ranked 21,628 genes in descending order by calculating the log_2_FC for each gene. Second, the GSEA was performed on these ranked genes using the KEGG gene sets. The enrichment KEGG pathways with *p* < 0.05 and |Normalized Enrichment Score (NES)| > 1 were considered significant (Zheng et al., 2022). NES was the primary statistic to examine the enrichment results, the value indicates the relationships between enrichment pathways and key genes. Herein, the intersection of enrichment KEGG pathways of each key gene was determined as the potential pathway in DCM with HF. Subsequently, we carried out Pearson’s correlation analysis between each key gene and the potential pathway-related genes. |*R*| > 0.3 and *p* < 0.05 was the cutoff criteria. The intersection of pathway genes significantly correlated with each key gene was considered important and used for further analysis. Next, the Pathview online tool (https://pathview.uncc.edu/home) (Luo et al., 2017) was used to perform Z-score normalization on the expressions of these intersection genes between control and DCM with HF groups and projected their relative expression values onto the KEGG pathway.

### Statistical analysis

Statistical analysis was conducted using R programming language (version 4.2.1). Wilcoxon test was performed to analyze the differential expression of key genes in DCM with HF group versus control group. The diagnostic ability of each key gene was evaluated by the AUC score. *P* < 0.05 was considered statistically significant.

## Results

### Identification of DEGs in DCM with HF

**FIGURE 1:**
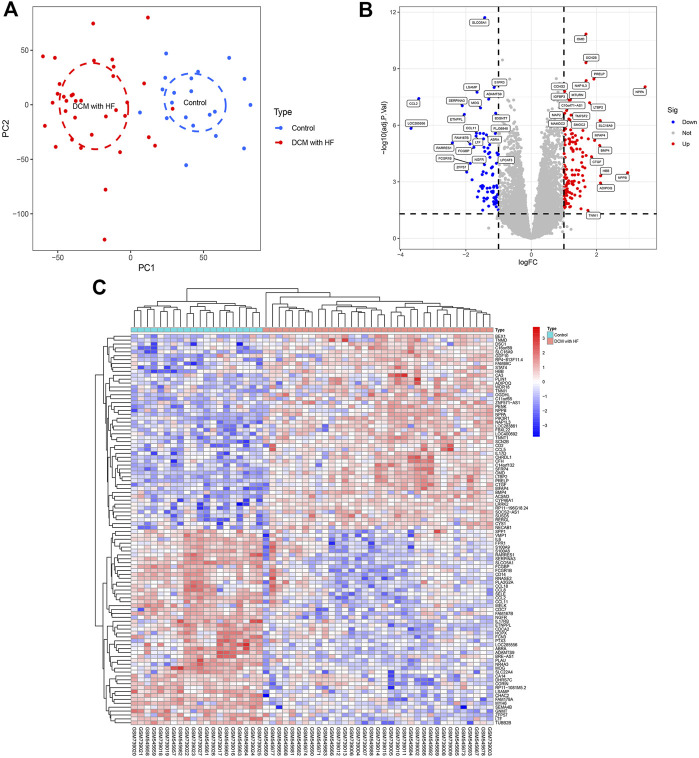
Data processing and identification of DEGs. **(A)** PCA score plot of the training group dataset after batch removal showing the difference between control and DCM with HF groups. **(B)** Volcano plot of DEGs. Red dots represent up-regulated genes and blue dots represent down-regulated genes. **(C)** Heatmap displaying a differential expression of the top 50 up-regulated genes and top 50 down-regulated genes.

The gene expression matrices of the training group were obtained after data cleaning, normalization, batch effect removal, and merging, including 20 normal myocardial samples (control group) and 35 myocardial samples derived from DCM patients with HF (DCM with HF group). The PCA score plot showed the relatively consistent clustering of two groups from two components (Figure 1A), which can be used in subsequent analysis due to between-group differences. A total of 218 DEGs (135 up-regulated genes and 83 down-regulated genes) were screened under the threshold (|log_2_FC| > 1 and adjusted *p* < 0.05). The Volcano plot of DEGs was then plotted using the log_2_FC and -log10 (adjusted *p*) (Figure 1B). The top 50 up-regulated genes and the top 50 down-regulated genes were shown in the heatmap (Figure 1C).

### Inference of biological functions, pathways, and diseases for up-regulated DEGs

**FIGURE 2:**
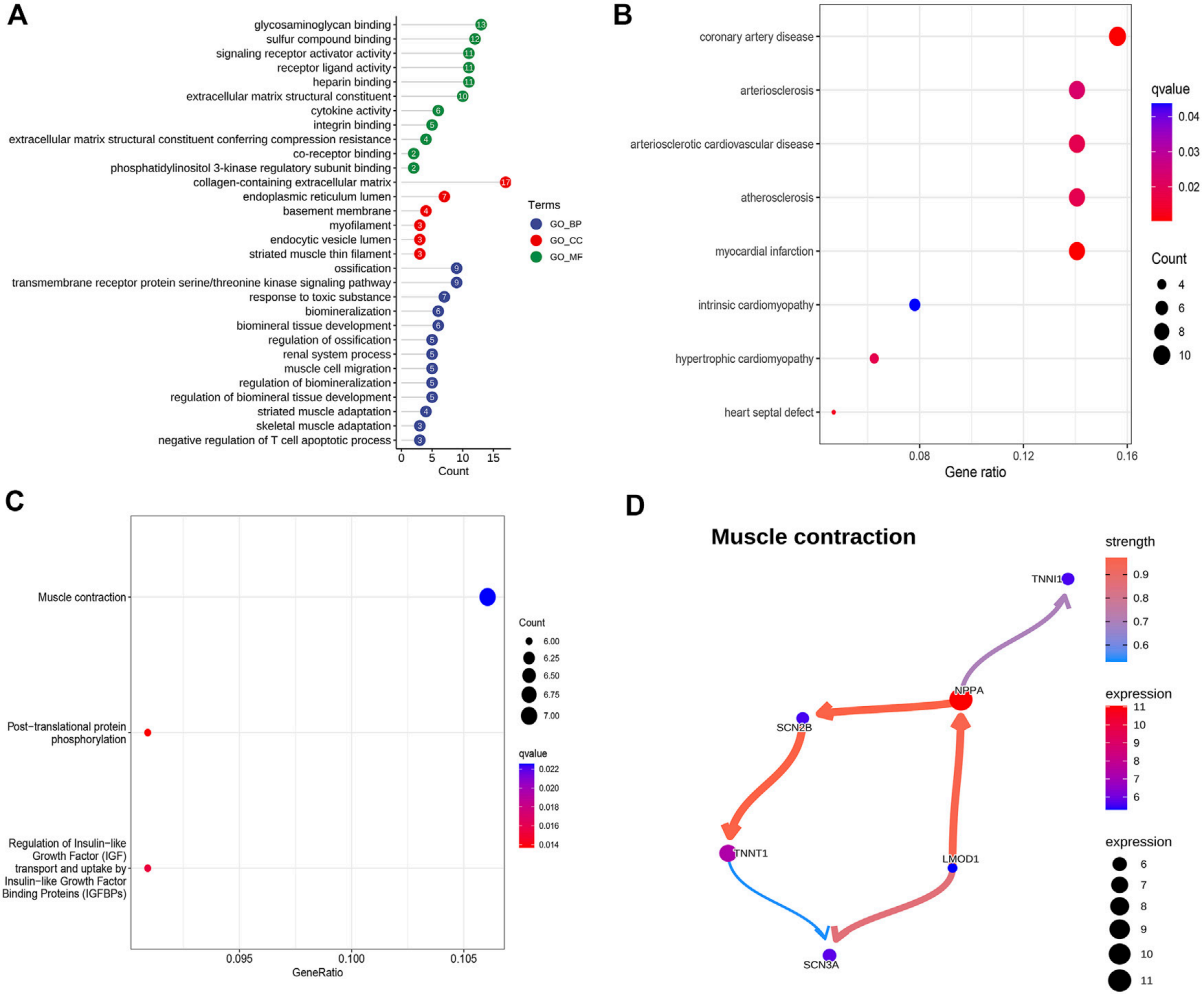
Potential biological function, pathways, and associated diseases of up-regulated DEGs. **(A)** GO enrichment analysis of up-regulated DEGs. **(B)** DO enrichment analysis of up-regulated DEGs. **(C)** KEGG enrichment analysis of up-regulated DEGs. **(D)** Bayesian network plot showing the regulatory relationships of genes in muscle contraction.

The GO, KEGG, and DO enrichment analyses were conducted to annotate the potential functions, pathways, and diseases of 135 up-regulated DEGs, respectively. As shown in Figure 2A, the enrichment GO terms of biological process (BP) included muscle cell migration, striated muscle adaptation, and skeletal muscle adaptation; the enrichment GO terms of cellular component (CC) included collagen−containing extracellular matrix, myofilament, and striated muscle thin filament; the enrichment GO terms of molecular function (MF) included integrin binding, cytokine activity, and extracellular matrix structural constituent. DO enrichment analysis showed that the DEGs were associated with many heart diseases, such as arteriosclerotic cardiovascular disease, coronary artery disease, and myocardial infarction (Figure 2B). Additionally, the enriched KEGG pathways mainly contained muscle contraction, post-translational protein phosphorylation, and regulation of Insulin-like Growth Factor (IGF) transport and uptake by Insulin-like Growth Factor Binding Proteins (IGFBPs) (Figure 2C).The BN plot further demonstrated the enriched gene interactions in muscle contraction (Figure 2D). Furthermore, NPPA acted as the core gene with higher expression and more edges, indicating its crucial role in muscle contraction.

### Weighted gene co-expression network construction

**FIGURE 3:**
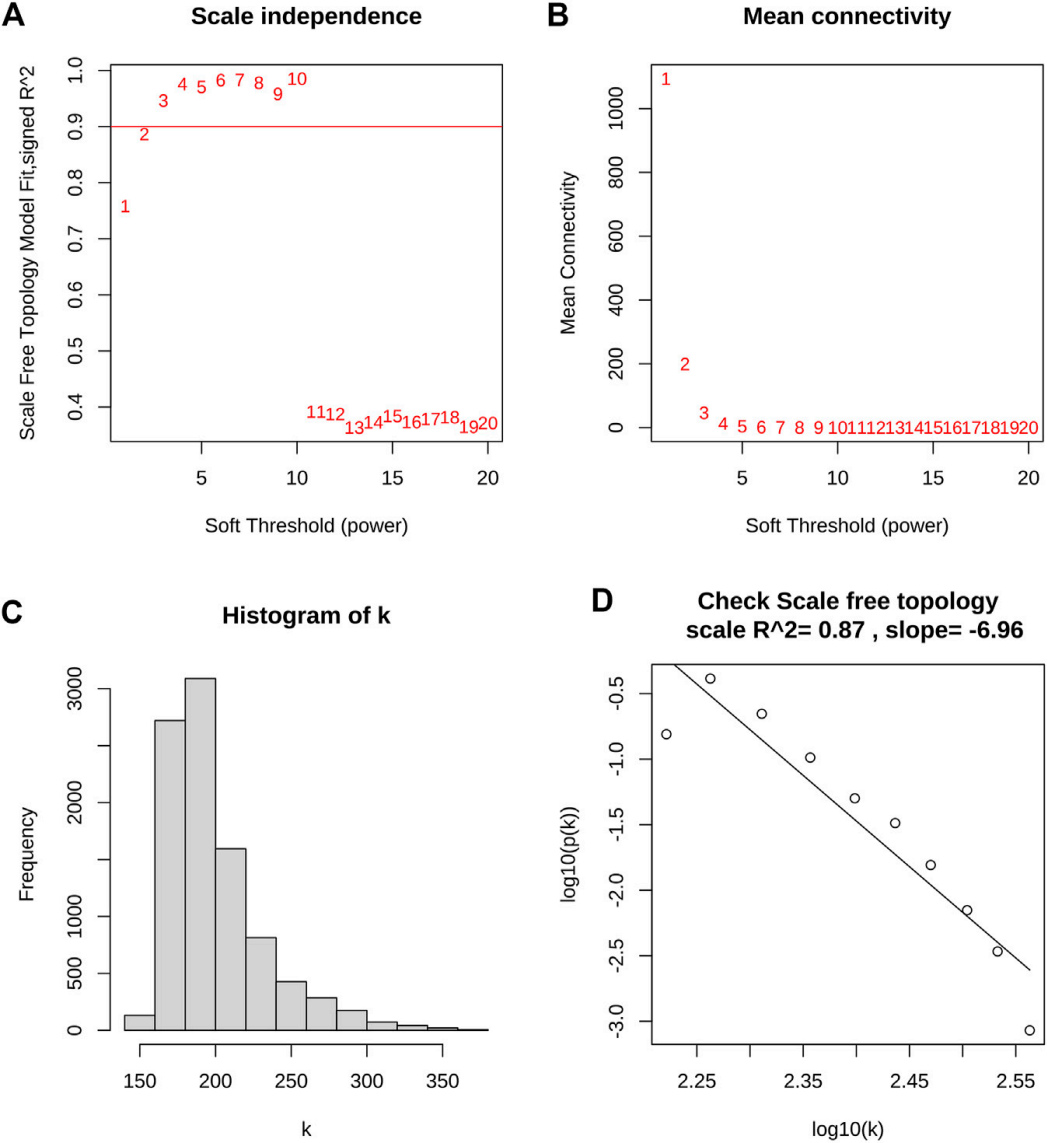
Determination of optimal soft threshold power for scale-free topology. **(A)** The scale-free topology fit index (*R* ^2^) for soft threshold power (*β*) from 1 to 20. **(B)** The mean connectivity (k) for *β* from 1 to 20. **(C)** Histogram of connectivity frequency. **(D)** The scale-free topology *R* ^2^ between log_10_(k) and log_10_ (p(k)) when *β* = 2.

**FIGURE 4:**
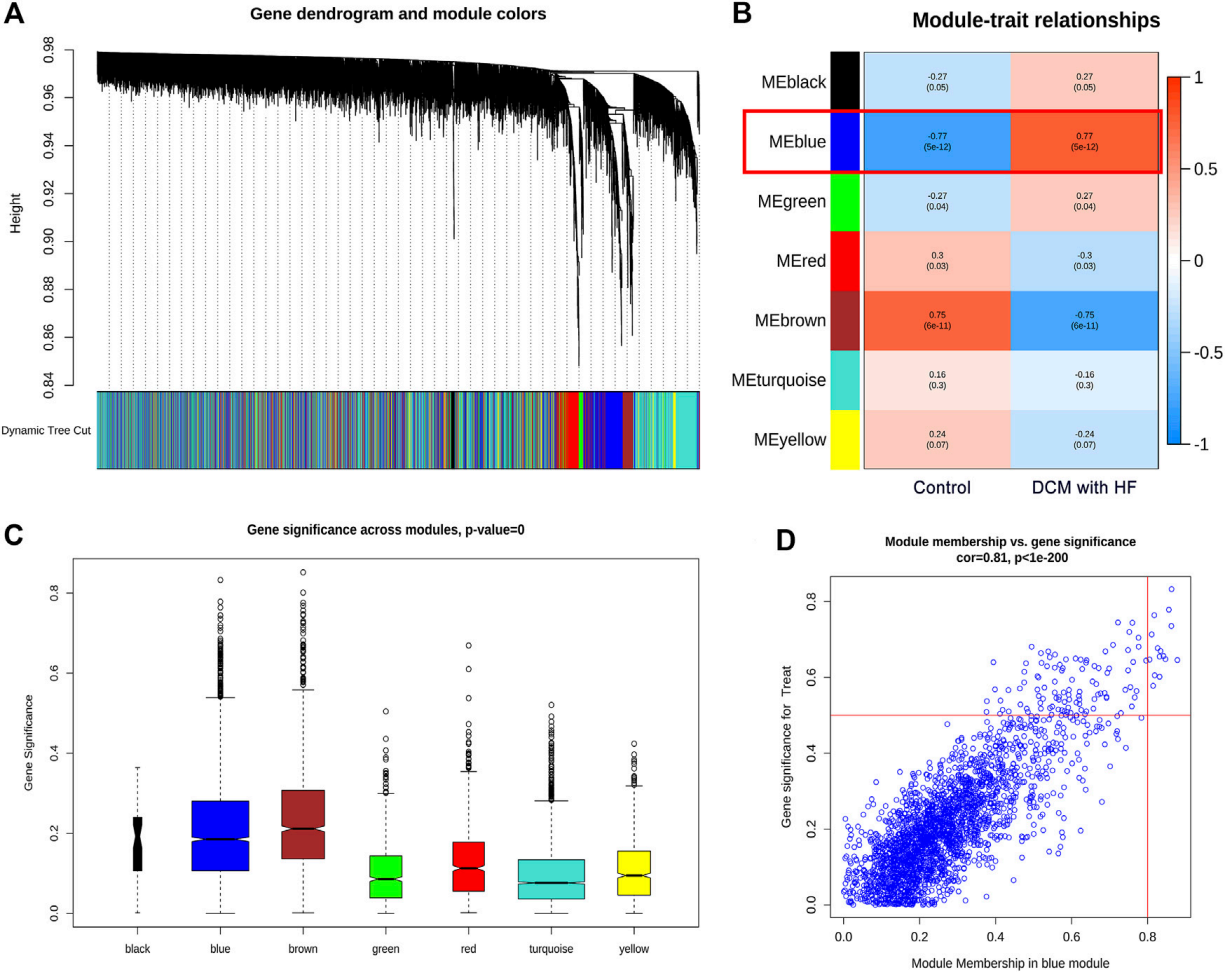
Identification of driver genes in module related to DCM with HF. **(A)** Dendrogram of all genes with dissimilarity clustered on topological overlap. **(B)** Correlation of seven modules and two traits (control and DCM with HF). The blue module was significantly correlated with DCM with HF (*R* = 0.77, *p* < 0.001). **(C)** Boxplot of Gene Significance (GS) distribution across seven modules. **(D)** Scatterplot of Module Membership (MM) in the blue module versus GS for DCM with HF.

WGCNA was utilized to construct the gene co-expression network and identify the trait-correlated modules in the training group. First, the similarity matrix was calculated and then transformed into the adjacent matrix via the optimal soft threshold power (*β* = 2) (Figures 3A,B). Besides, the inverse relationship of p(k) and k was observed (Figure 3C), and the scale-free topology *R* ^2^ of log10(k) and log10 (p(k)) was 0.87 when *β* = 2 (Figure 3D), which illustrated *β* = 2 was suitable for constructing a scale-free topology. Second, the TOM was converted from the adjacent matrix and turned into the dissTOM (dissTOM = 1-TOM) for hierarchical clustering. Dynamic tree shearing clustered the similarity genes based on the topological overlap and then divided them into various modules (Figure 4A). Third, we related the modules to clinical trait and further obtained the driver genes in the strongest positive correlation module. As shown in Figure 4B, we ultimately identified the ME blue module strongly positively related to DCM with HF (*R* = 0.77, *p* < 0.001), containing 2,411 genes. Fourth, the significant GS distribution across the seven modules was observed (Figure 4C). Additionally, the intramodular analysis demonstrated the close relationship between MM and GS of the ME blue module (*R* = 0.81, *p* < 0.001; Figure 4D), thus relieving the module genes highly associated with DCM with HF. Finally, a total of 15 genes with GS > 0.5 and MM > 0.8 (C14orf132, CFH, COL8A1, CTGF, ETNPPL, FIBIN, FRZB, ITGBL1, LTBP2, MFAP4, NPPA, NRK, OMD, PRELP, SFRP4) were identified as driver genes in the ME blue module.

### Construction and validation of BPNN on 13 feature genes

**FIGURE 5:**
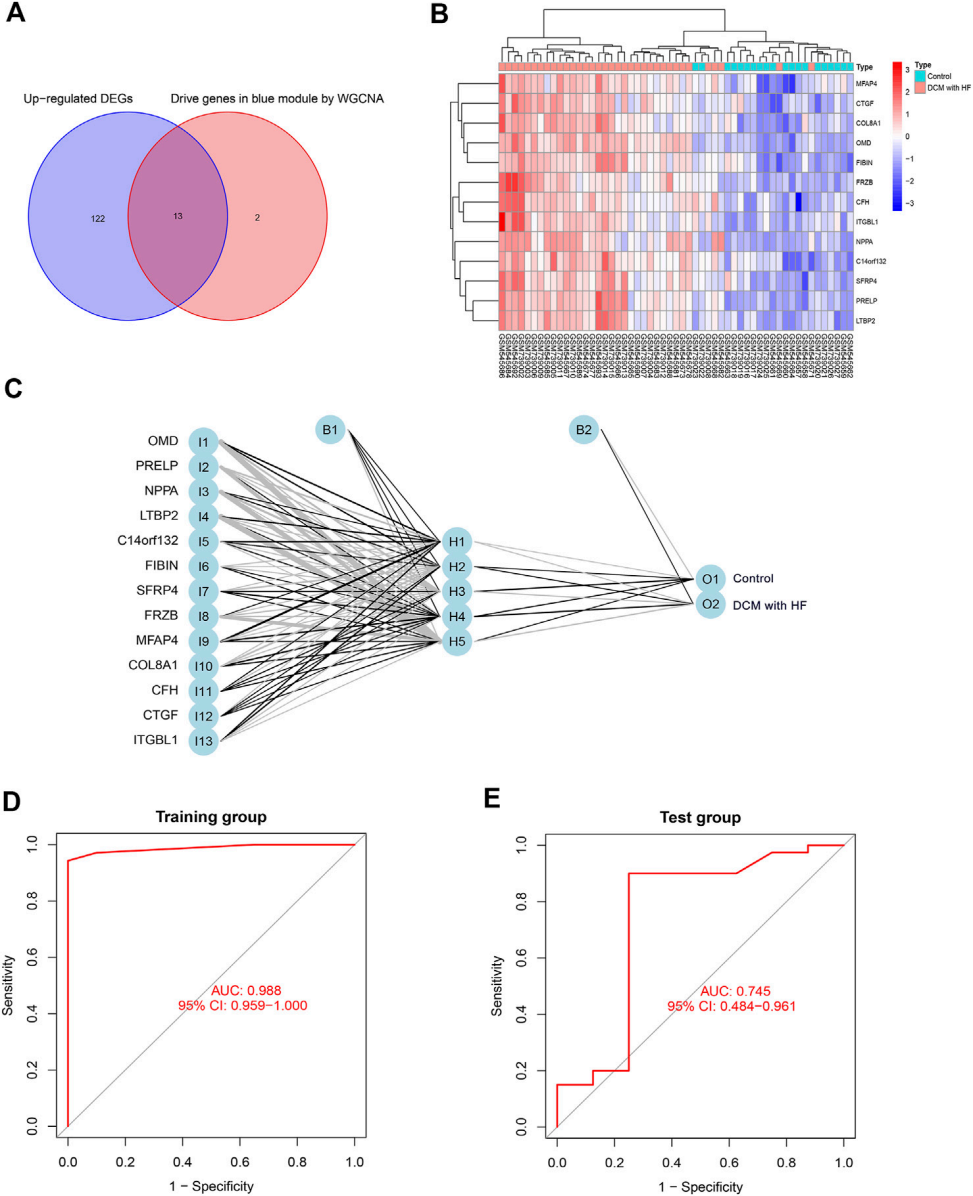
Construction and validation of BPNN on 13 feature genes. **(A)** The intersection of 135 up-regulated genes and 15 driver genes in the blue module as feature genes. **(B)** Expression heatmap of 13 feature genes in control and DCM with HF groups. **(C)** BPNN constructed with 13 neurons in the input layer, five neurons in the hidden layer, and two neurons in the output layer. ROC curve for evaluating classification accuracy in the training group **(D)** and test group **(E)**. I, input layer; H, hidden layer; O, output layer; B, bias.

We selected the intersection of 135 up-regulated DEGs and 15 driver genes in the ME blue module as the feature genes of DCM with HF, with a total of 13 genes (OMD, PRELP, NPPA, LTBP2, C14orf132, FIBIN, SFRP4, FRZB, MFAP4, COL8A1, CFH, CTGF, ITGBL1) (Figure 5A). These 13 genes were previously proved to participate in muscle contraction, striated muscle adaptation, and extracellular matrix structural constituent (Figures 2A,B), indicating their potential crucial roles in DCM with HF. Figure 5B showed the differential expressions of feature genes between control and DCM with HF groups. Subsequently, a BPNN with 13 neurons in the input layer (13 feature genes), five neurons in the hidden layer, and two neurons in the output layer (classification numbers: control or DCM with HF) was constructed in the training group and then validated in the test group (Figure 5C). The BPNN showed prediction accuracy with a high AUC score of 0.988 in the training group (Figure 5D). However, the predictive power of BNPP decreased significantly in the test group compared to the training group, merely achieving an AUC score of 0.745 (Figure 5E). These results suggested that the collinearity between the screened 13 feature genes resulted in the constructed BPNN over-fitting for general prediction (Supplementary Figure S2). Thus, feature selection was crucial for further obtaining the key genes for the diagnosis of DCM with HF.

### NPPA, OMD, and PRELP were the key genes for DCM with HF

**FIGURE 6:**
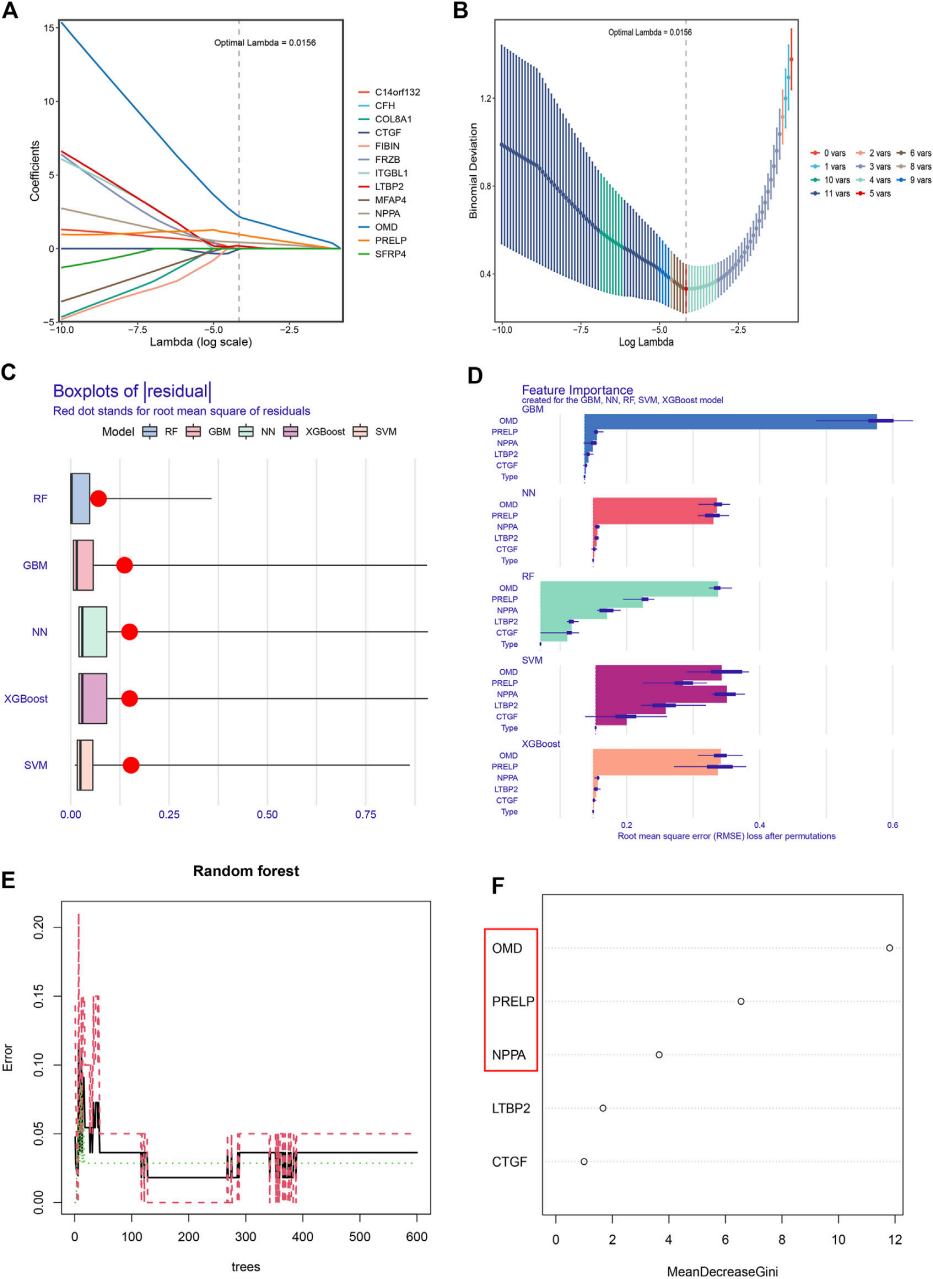
Obtaining the key genes for the diagnosis of DCM with HF. **(A)** The coefficients of 13 feature genes shown by log(λ). **(B)** The relationship between binomial deviation and log(λ) by 10-fold cross-validation (CV). **(C)** Residual diagram of Random Forest (RF), Gradient Boosting Machine (GBM), Neural Network (NN), eXtreme Gradient Boosting (XGBoost), and Support Vector Machine (SVM) classification. **(D)** Root Mean Square Error (RMSE) loss after feature removal from models. **(E)** The effect of decision tree number on CV error of RF classifier. Green, red, and black curves represent the error of control, DCM with HF, and total groups, respectively. **(F)** The mean decrease Gini coefficients of genes in the RF classifier. OMD, PRELP, and NPPA were the key genes with mean decrease Gini larger than 2.

To narrow down the range of feature genes, Lasso within the training group was first performed. As shown in Figure 6A, Lasso retained five genes from 13 feature genes under the optimal log(λ) as 0.0156. Through the 10-fold cross-validation, the minimum binomial deviation with optimal log(λ) was observed, indicating the five genes were optimally selected by Lasso (Figure 6B). Second, five machine learning models (RF, GBM, NN, XGBoost, and SVM) were established on the five LASSO-selected genes and validated their classification performances based on a series of indexes (residual, Brier score, AUC score, NB) in the training and test groups. Strikingly, the RF model showed robust classification ability, with the lowest residual (Figure 6C) and Brier score (Supplementary Table S1), highest AUC achieving 1 (Supplementary Figures S3A,C), and NB under threshold probability (Supplementary Figures S3B,D) in the training and test groups. Thus, the RF classifier was considered the most suitable performance classifier for further feature selection. Moreover, the importance of feature genes selected from five classifiers was illustrated by Root Mean Square Error (RMSE) loss (Figure 6D). Third, we constructed the RF model with 400 decision trees (Figure 6E), and three features with mean decrease Gini coefficients larger than 2 (OMD, PRELP, NPPA) were selected as key genes for diagnosing DCM with HF (Figure 6F).

### NPPA, OMD, and PRELP were up-regulated with diagnostic abilities in DCM with HF

**FIGURE 7:**
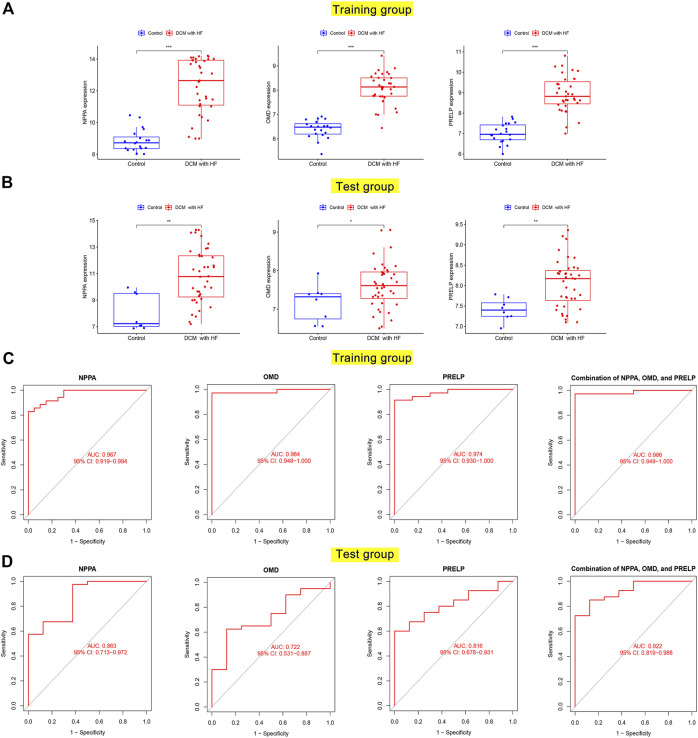
Differential expression and diagnostic accuracy of key genes in the training and test groups. The mRNA expression levels of NPPA, OMD, and PRELP between normal hearts and damaged hearts of DCM with HF in the training group **(A)** and test group **(B)**. ROC curves for evaluating diagnostic accuracy of NPPA, OMD, and PRELP in the training group **(C)** and test group **(D)**.

To test whether NPPA, OMD, and PRELP could serve as the diagnostic biomarkers of DCM with HF, their mRNA expression levels and diagnostic abilities were then analyzed. As shown in Figures 7A,B, the over-expression levels of NPPA, OMD, and PRELP were observed in DCM with HF in the training group and test group. Furthermore, the AUC scores of NPPA (AUC = 0.967), OMD (AUC = 0.984), PRELP (AUC = 0.974), and their combination (AUC = 0.986) for diagnosing DCM with HF were all larger than 0.9 in the training group (Figure 7C). The AUC scores of NPPA (AUC = 0.863), OMD (AUC = 0.722), and PRELP (AUC = 0.816) decreased in the test group (Figure 7D). However, the AUC score of the combination of NPPA, OMD, and PRELP markedly achieved 0.922 within the test group. These results suggested NPPA, OMD, and PRELP be promising diagnostic biomarkers for DCM with HF. To measure the combined diagnostic accuracies of NPPA, OMD, and PRELP, a logistic regression model was constructed and displayed in the nomogram (Supplementary Figure S4). Interestingly, the combination of NPPA, OMD, and PRELP improved the diagnostic effect within the training and test groups. We also observed the heart muscle-specific expressions of NPPA, OMD, and PRELP, and their extracellular locations were all predicted to be secreted in the HPA database (Supplementary Figure S5), which provided strong evidence that NPPA, OMD, and PRELP served promising biomarkers of DCM with HF.

### Target therapeutic drug prediction for NPPA

Herein, we used DSigDB to predict the potential therapeutic drugs associated with NPPA, OMD, and PRELP. A total of 17 target drugs related to NPPA were finally predicted, but not associated with OMD and PRELP. And the specific information on the top 10 predicted drugs was shown in Supplementary Table S2. Among these drugs, the antihypertensive efficacy of alprostadil, labetalol, felodipine, and irbesartan has been reported (Johnson and Fugman, 1983; MacCarthy and Bloomfield, 1983; Cattaneo et al., 2003). Additionally, previous studies have shown that irbesartan, furosemide, and Bonuten can treat HF (Johnson and Fugman, 1983; Swedberg et al., 1987; Thompson et al., 1999). Several pieces of evidence have supported that these predictive drugs targeting NPPA could cure some cardiovascular disorders such as hypertension and HF, thus indicating their value in the treatment of DCM with HF.

### Single-cell RNA-seq analysis revealed the over-expression patterns of NPPA, OMD, and PRELP were stable in DCM patients

**FIGURE 8:**
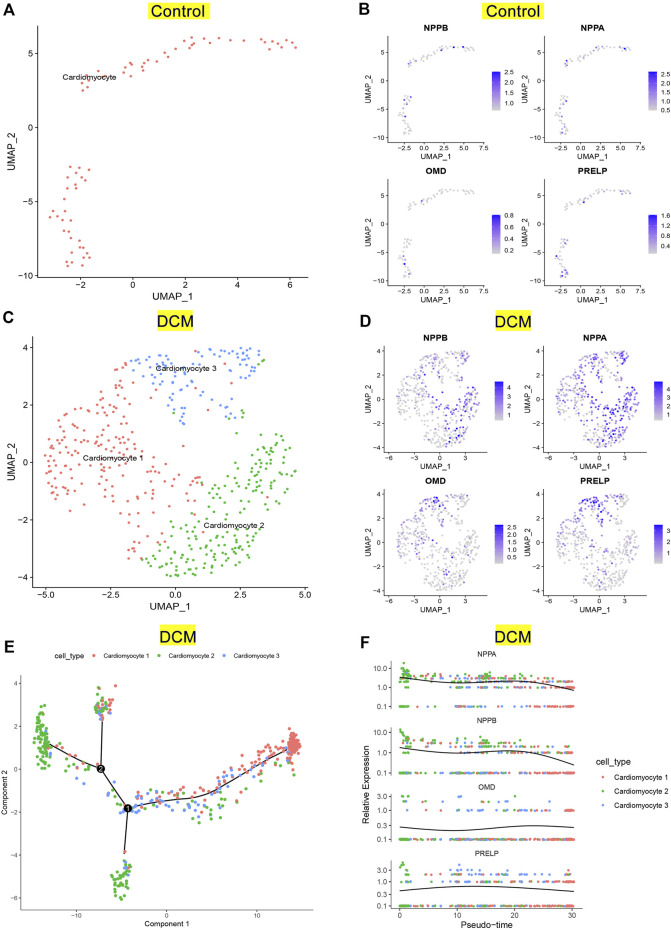
Verification of key genes expression pattern on single-cell RNA-seq analysis. **(A)** UMAP projection of distinct normal cardiomyocytes in GSE95140. **(B)** Distribution of NPPB, NPPA, OMD, and PRELP in distinct normal cardiomyocytes. **(C)** UMAP projection showing three types of cardiomyocytes of DCM patients in GSE95140. **(D)** Distribution of NPPB, NPPA, OMD, and PRELP in distinct cardiomyocytes of DCM. **(E)** Pseudotime-ordered analysis of cardiomyocytes of DCM. **(F)** Dynamic expression of NPPB, NPPA, OMD, and PRELP by the Pseudotime.

**FIGURE 9:**
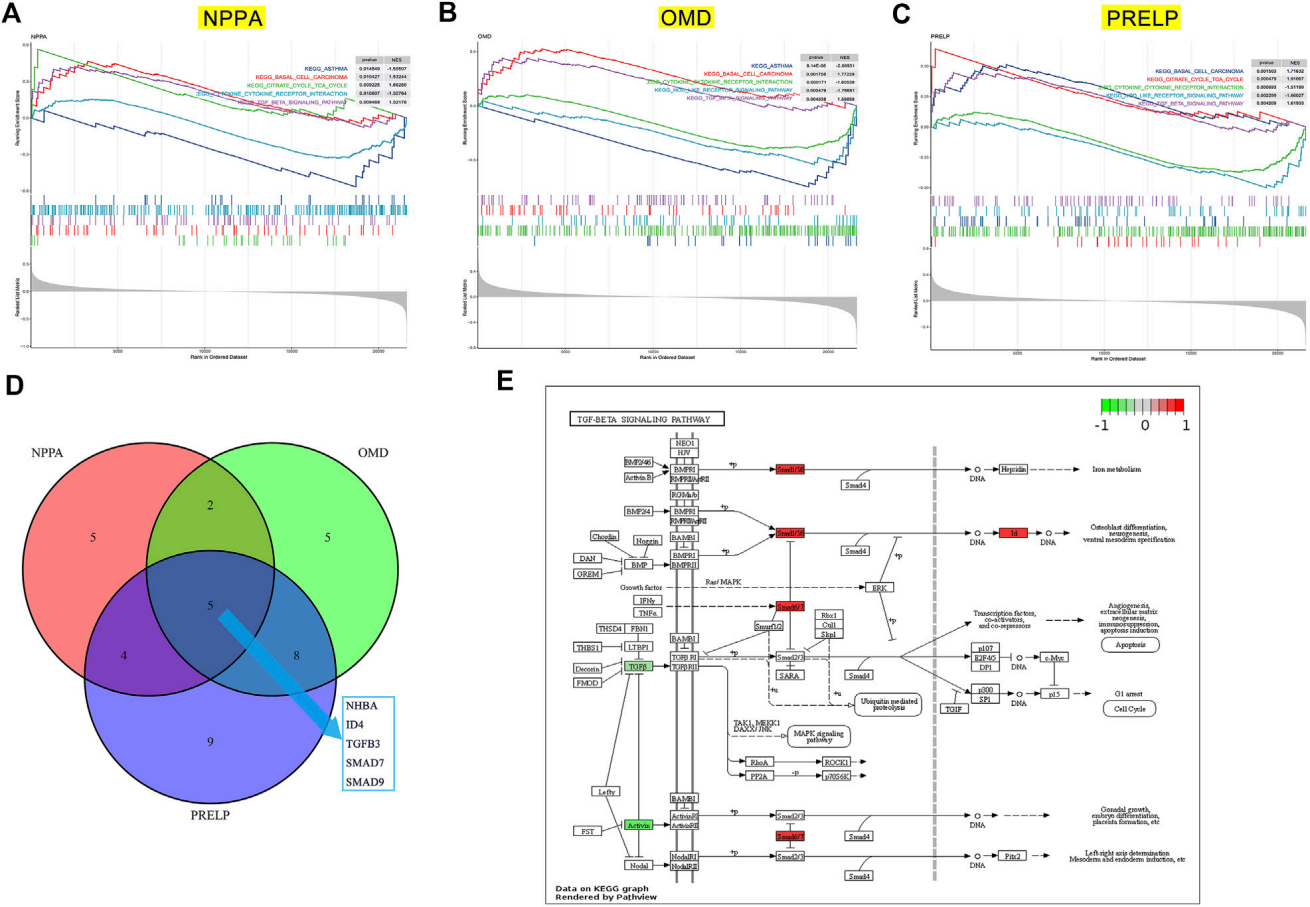
The potential pathway regulated by key genes in the development of DCM with HF. GSEA on low- and high-expression groups of NPPA **(A)**, OMD **(B)**, and PRELP **(C)**. **(D)** Intersection of TGF-β pathway genes significantly correlated with NPPA, OMD, and PRELP. **(E)** The relative expression of INHBA, ID4, TGFB3, SMAD7, and SMAD9 in TGF-β signaling pathway.

To investigate the expression patterns of NPPA, OMD, and PRELP in DCM patients, we conducted single-cell RNA-seq analysis on GSE95140. The quality control, data cleaning, and PC selection were shown in Supplementary Figure S6. The entire cell profiles of the control group and DCM group were classified into 2 clusters (Figure 8A) and 3 clusters (Figure 8C) and projected onto UMAP plots, respectively. We identified the cell type using specific gene markers (NNT2 and MYH7 for cardiomyocyte; DCN and COL1A1 for fibroblast; CDH5 and VWF for endothelial cell) in the control and DCM groups (Supplementary Figure S7). We annotated one cardiomyocyte type and three distinct cardiomyocyte types in the control group and DCM group, respectively (Figures 8A,C). Then, we analyzed the expression of NPPA, OMD, PRELP, and NPPB in cardiomyocytes of control and DCM groups. As shown in Figures 9B,D, higher expression levels of NPPA, OMD, PRELP, and NPPB were observed in most cardiomyocytes in DCM compared to the control group. Interestingly, we noticed that type 2 of the DCM group showed high expression of NPPA and NPPB, suggesting that cardiomyocytes with higher degrees of HF in these types. We also performed Pseudotime-ordered analysis on the DCM group to explore the expression changes of NPPA, OMD, PRELP, and NPPB during the cardiomyocyte transitions along the Pseudotime. Figure 9E showed the differentiation trajectory path of cardiomyocytes ordered by different types. Figure 9F further displayed the stable expression patterns of NPPA, OMD, PRELP, and NPPB in diverse cardiomyocytes by Pseudotime. Notably, the dynamic expression changes of NPPA and NPPB were similar, indicating an expression linkage between NPPA and NPPB in DCM. Overall, these results showed NPPA, OMD, and PRELP were enriched in cardiomyocytes of DCM and displayed stable expression patterns, which fluctuated slightly with differential cell states.

### NPPA, OMD, and PRELP may participate in TGF-β signaling pathway in DCM with HF

To gain insight into the potential pathways of NPPA, OMD, and PRELP, GSEA was performed for each gene. As shown in Figure 9A, the top three positively significant KEGG pathways for NPPA were “citrate cycle (TCA cycle)”, “basal cell carcinoma”, and “TGF-β signaling pathway”. The top two positively significant KEGG pathways for OMD were enriched (Figure 9B), including “basal cell carcinoma” and “TGF-β signaling pathway”. The top three positively significant KEGG pathways for PRELP shown in Figure 9C were “citrate cycle (TCA cycle)”, “basal cell carcinoma”, and “TGF-β signaling pathway”. Interestingly, the terms “basal cell carcinoma” and “TGF-β signaling pathway” were enriched in the KEGG pathways of NPPA, OMD, and PRELP, and acted as the positive pathways. The TGF-β signaling pathway was considered the most likely mechanism involved by NPPA, OMD, and PRELP. We then analyzed Pearson’s correlation of NPPA, OMD, and PRELP and the TGF-β signaling pathway gene sets (Supplementary Table S3), respectively. And 16 NPPA-correlated, 20 OMD-correlated, and 26 PRELP-correlated genes were obtained (Supplementary Table S4). The intersections of these genes were INHBA, ID4, TGFB3, SMAD7, and SMAD9 (Figure 9D). Then, the expression levels of INHBA, ID4, TGFB3, SMAD7, and SMAD9 between control and DCM with HF groups were normalized and visualized in the TGF-β signaling pathway (Figure 9E). Additionally, we observed that ID4, SMAD7, and SMAD9 were up-regulated in the TGF-β signaling pathway. While INHBA and TGFB3 were down-regulated. Accordingly, we inferred that the TGF-β signaling pathway positively associated with NPPA, OMD, and PRELP might play a crucial role in the process of DCM with HF.

## Discussion

The pathogenesis of DCM with HF has remained unclear, mostly because of the heterogeneous etiology and clinical presentation (Schultheiss et al., 2019). Considering that the progressive condition of DCM leads to impaired contractility, preventing or treating HF is currently the first-line therapy for DCM patients (Schultheiss et al., 2019; Mahmaljy et al., 2022). For DCM patients with end-stage HF, the sole feasible treatment is heart transplantation (Iwata et al., 2020). Accordingly, identifying underlying genes and mechanisms of DCM with HF may help improve the early diagnosis and novel treatment. In this study, we conducted a systemic bioinformatics analysis on mRNA-seq and scRNA-seq data from DCM patients with HF. The promising diagnosis biomarkers were screened through WGCNA and machine learning, and the regulated potential pathway in the development of DCM with HF were further identified.

To our knowledge, this is the first attempt to combine WGCNA with machine learning to explore specific biomarkers to distinguish DCM patients with HF from the control group. The newly developed bioinformatics method, CBNplot, was utilized for exploring the pathway and gene interaction on Bayesian network inference (Sato et al., 2022). In our research, we first screened 218 DEGs (135 up-regulated genes and 83 down-regulated genes) between control and DCM with HF groups. Then, we performed GO, KEGG, and DO enrichment analyses on 135 up-regulated DEGs. The results showed that the enriched GO terms and KEGG pathways were related to muscle cell functions or activities, such as muscle cell migration, myofilament, and muscle contraction. And many heart diseases such as arteriosclerotic cardiovascular disease, coronary artery disease, and myocardial infarction were enriched. Previous studies have shown dysfunctional myocardium in DCM patients with HF (Schwinger et al., 1992). Thus, 135 up-regulated DGEs might involve in the pathological process of DCM with HF, were obtained for further selection. In addition, WGCNA was conducted to identify the gene module (ME blue module) that is highly positively related to DCM with HF (*R* = 0.77, *p* < 0.001) based on gene co-expression pattern. We then obtained 15 driver genes with higher GS and MM (C14orf132, CFH, COL8A1, CTGF, ETNPPL, FIBIN, FRZB, ITGBL1, LTBP2, MFAP4, NPPA, NRK, OMD, PRELP, SFRP4) from the ME blue module. The intersection of 15 diver genes and 135 up-regulated genes, OMD, PRELP, NPPA, LTBP2, C14orf132, FIBIN, SFRP4, FRZB, MFAP4, COL8A1, CFH, CTGF, and ITGBL1, were then selected as 13 features to construct a BNPP classifier in the training and test groups. The BPNN established with 13 neurons in the input layer, five neurons in the hidden layer, and two neurons in the output layer showed excellent classification performance in the training group (AUC = 0.988), but the performance in the test group was poor (AUC = 0.745). The results suggested that the BNPP on 13 feature genes was not suitable as an over-fitting model to distinguish DCM patients with HF from normal. Col-linearity between these 13 feature genes may be the reason why the over-fitting model was constructed (Supplementary Figure 2). Accordingly, further feature selection was needed to identify the key genes of DCM with HF.

Herein, we first performed LASSO selection to narrow the range of feature genes and identified five genes with non-zero coefficients (OMD, PRELP, NPPA, LTBP2, and CTGF). Next, five well-established machine learning algorithms, including RF, GBM, NN, XGBoost, and SVM, were applied as classifiers based on the LASSO-selected genes. We then assessed their classification performances within the training and test groups by residual, AUC score, Brier score, and NB. And RF performed with the lowest residual and Brier score, along with the highest AUC and NB was considered the best-performing classifier. Thus, three significant explanatory features (OMD, PRELP, and NPPA) in RF were selected as key genes for DCM with HF. To test whether OMD, PRELP, and NPPA could serve as diagnostic biomarkers, we analyzed their expression levels and diagnostic values in DCM with HF. Boxplots demonstrated the up-regulation of OMD, PRELP, and NPPA in the DCM with HF group. Within the training and test groups, OMD, PRELP, and NPPA had certain diagnostic values (all AUC >0.7), of which PRELP and NPPA may have significant diagnostic abilities (all AUC >0.8). We also found that the combination of OMD, PRELP, and NPPA could efficiently distinguish DCM patients with HF from normal, with AUC = 0.986 in the training group and AUC = 0.922 in the test group. These results suggested that OMD, PRELP, and NPPA may be promising diagnostic biomarkers, along with high expression levels in DCM with HF. Additionally, the heart muscle-specific and secreted expressions observed in the HPA database also demonstrated the potential application of OMD, PRELP, and NPPA as diagnostic biomarkers. Additionally, DSigDB was used to predict the therapeutic drugs targeting NPPA for finding the potential effective drug therapy of DCM with HF. Several identified drugs, including alprostadil, labetalol, felodipine, irbesartan, furosemide, and Bonuten, have been proven to cure cardiovascular diseases such as hypertension and HF. These results indicated their potential application in treating DCM with HF. However, the specific role of the predicted drugs in DCM with HF still needed further experimental validations *in vivo* and *in vitro*.

To further investigate the expression distributions and changes of OMD, PRELP, and NPPA, single-cell RNA-seq analysis was conducted to evaluate their expressions in cardiomyocytes derived from DCM patients. We first delineated the cardiomyocyte distributions in the control and DCM groups using UMAP plots, respectively. Leveraging gene markers (cardiomyocyte, fibroblast, and endothelial cell) from CellMarker2.0 database, distinct cardiomyocyte types were then annotated and identified. Subsequently, the high expression patterns of NPPA, OMD, and PRELP were observed in cardiomyocytes of DCM. We also explored the dynamic expression patterns of NPPA, OMD, and PRELP during the cardiomyocyte transitions across the Pseudotime. It was shown that the expression fluctuations of NPPA, OMD, and PRELP demonstrated stable expression patterns in cardiomyocyte differentiation ordered by the Pseudotime. Moreover, we found that NPPA expression pattern in DCM was similar to NPPB, a classical biomarker of HF. In summary, these results showed that the over-expression patterns of NPPA, OMD, and PRELP were relatively stable regardless of cardiomyocyte types or states, which suggests the large potential for serving diagnostic biomarkers.

Among the identified three genes of DCM with HF, NPPA has already been known to involved in cardiomyopathies, while reports on OMD and PRELP are limited. Atrial Natriuretic Peptide (ANP) encoded by NPPA belongs to the natriuretic peptide family and has been developed to be diagnostic biomarkers of HF (Goetze et al., 2020), which also demonstrated NPPA can be a positive biomarker control to validate our analysis accuracy. OMD encoded a leucine-rich keratin sulfate proteoglycan located in the extracellular matrix (ECM), which participated in cell adhesion and tissue mineralization (Skenteris et al., 2022). A recent study unraveled that OMD may be an early plasma biomarker of cardiovascular calcification (Skenteris et al., 2022). Furthermore, the association between OMD and cardiovascular risk factors was observed in a large aptamer-based proteomic profiling research (Ngo et al., 2021). Interestingly, a bioinformatics study identified OMD as the promising biomarker and therapeutic target of hypertrophic cardiomyopathy (Guo et al., 2021). PRELP belongs to the leucine-rich repeat (LRR) family, and encodes a leucine-rich repeat protein, which exists in ECM of connective tissues (Bengtsson et al., 1995). The role of PRELP in cardiac extracellular matrix remodeling has been demonstrated in proteomics research on the ischemia/reperfusion injury model (Barallobre-Barreiro et al., 2012). Zhang et al. recently reported that PRELP led to myocardial fibrosis and ventricular remodeling following acute myocardial infarction, which highlights the role of PRELP in cardiovascular disease (Zhang et al., 2022). Overall, previous studies have elucidated the pathological roles or diagnostic values of NPPA, OMD, and PRELP in different cardiovascular diseases, but have not yet involved DCM with HF.

To explore the common underlying mechanism of NPPA, OMD, and PRELP in DCM with HF, we performed GSEA based on KEGG gene sets. The GSEA results indicated that the KEGG pathways of NPPA, OMD, and PRELP were all enriched in the TGF-β signaling pathway. Thus, TGF-β signaling pathway might serve as a crucial mechanism in DCM with HF. Next, we analyzed the correlation between each key gene (NPPA, OMD, PRELP) and the TGF-β signaling pathway-related genes. INHBA, ID4, TGFB3, SMAD7, and SMAD9, the intersection of the NPPA-correlated, OMD-correlated, and PRELP-correlated genes, were considered the most likely affected genes in the TGF-β signaling pathway. And the up-regulation of ID4, SMAD7, and SMAD9 and down-regulation of INHBA, TGFB3 were observed in DCM with HF group. Evolving studies demonstrate that TGF-β signaling pathway is critically participated in HF and cardiac remodeling (Dobaczewski et al., 2011). Increased systemic and myocardial TGF-β levels were found in patients with DCM, suggesting the activation of TGF-β signaling pathway, which was consistent with our result (Dobaczewski et al., 2011). Thus, combining with the previous experimental evidence and our GSEA result, we speculated that the mediators of TGF-β signaling pathway may exert crucial actions through interacting with NPPA, OMD, and PRELP on the pathogenesis of DCM with HF.

However, there exists some shortcomings in our study. First, the five machine learning classifiers were established on small training data sets because of the limited databases of DCM with HF. Nevertheless, the best-performing classifier was comprehensively validated and then confirmed by a series of indexes. Accordingly, the selected features (key genes) were considered the most significant. Second, the up-regulation pattern of key genes (NPPA, OMD, and PRELP) needed further experimental verification, such as quantitative polymerase chain reaction or immunohistochemistry. Herein, to ensure the credibility of our analysis, external gene expression profiling and single-cell RNA seq data were used for validation. Third, combined GSEA and correlation analysis, we speculated that the TGF-β signaling pathway linking with NPPA, OMD, and PRELP might play a crucial role in DCM with HF. However, further functional studies are still needed to elucidate the specific roles and pathways of NPPA, OMD, and PRELP in DCM with HF.

In conclusion, we conducted a comprehensive bioinformatics study based on gene expression profiles and single-cell RNA sequencing datasets derived from DCM with HF patients. First, the combined analysis of WGCNA and RF classifier identified three key genes for the diagnosis of DCM with HF, out of which were NPPA, OMD, and PRELP. In addition, NPPA, OMD, and PRELP were increased in DCM with HF and showed excellent diagnostic abilities. And the up-regulated expression patterns of NPPA, OMD, and PRELP were stable, with minor fluctuation affected by differential cell states. Besides, the TGF-β signaling pathway, interacting with NPPA, OMD, and PRELP, may serve as a critical mechanism involvement in DCM with HF.

## Data Availability

Publicly available datasets were analyzed in this study. This data can be found here: Three gene expression profiling datasets of DCM with HF (GSE29819, GSE21610, GSE17800) and one single-cell RNA-sequencing dataset of DCM (GSE95140) were obtained from the GEO database (https://www.ncbi.nlm.nih.gov/geo/).

## References

[B1] Ai D. Wang Y. Li X. Pan H. (2020). Colorectal cancer prediction based on weighted gene co-expression network analysis and variational auto-encoder. Biomolecules 10 (9), 1207. 10.3390/biom10091207 32825264 PMC7563725

[B2] Alba A. C. Agoritsas T. Walsh M. Hanna S. Iorio A. Devereaux P. J. (2017). Discrimination and calibration of clinical prediction models: Users' guides to the medical literature. Jama 318 (14), 1377–1384. 10.1001/jama.2017.12126 29049590

[B3] Ameling S. Herda L. R. Hammer E. Steil L. Teumer A. Trimpert C. (2013). Myocardial gene expression profiles and cardiodepressant autoantibodies predict response of patients with dilated cardiomyopathy to immunoadsorption therapy. Eur. Heart J. 34 (9), 666–675. 10.1093/eurheartj/ehs330 23100283 PMC3584995

[B4] Barallobre-Barreiro J. Didangelos A. Schoendube F. A. Drozdov I. Yin X. Fernández-Caggiano M. (2012). Proteomics analysis of cardiac extracellular matrix remodeling in a porcine model of ischemia/reperfusion injury. Circulation 125 (6), 789–802. 10.1161/circulationaha.111.056952 22261194

[B5] Barth A. S. Kuner R. Buness A. Ruschhaupt M. Merk S. Zwermann L. (2006). Identification of a common gene expression signature in dilated cardiomyopathy across independent microarray studies. J. Am. Coll. Cardiol. 48 (8), 1610–1617. 10.1016/j.jacc.2006.07.026 17045896

[B6] Bengtsson E. Neame P. J. Heinegård D. Sommarin Y. (1995). The primary structure of a basic leucine-rich repeat protein, PRELP, found in connective tissues. J. Biol. Chem. 270 (43), 25639–25644. 10.1074/jbc.270.43.25639 7592739

[B7] Carniel E. Taylor M. R. Sinagra G. Di Lenarda A. Ku L. Fain P. R. (2005). Alpha-myosin heavy chain: A sarcomeric gene associated with dilated and hypertrophic phenotypes of cardiomyopathy. Circulation 112 (1), 54–59. 10.1161/circulationaha.104.507699 15998695

[B8] Cattaneo M. G. Pola S. Dehò V. Sanguini A. M. Vicentini L. M. (2003). Alprostadil suppresses angiogenesis *in vitro* and *in vivo* in the murine Matrigel plug assay. Br. J. Pharmacol. 138 (2), 377–385. 10.1038/sj.bjp.0705051 12540529 PMC1573673

[B9] Chen P. Long B. Xu Y. Wu W. Zhang S. (2018). Identification of crucial genes and pathways in human arrhythmogenic right ventricular cardiomyopathy by coexpression analysis. Front. Physiol. 9, 1778. 10.3389/fphys.2018.01778 30574098 PMC6291487

[B10] Dobaczewski M. Chen W. Frangogiannis N. G. (2011). Transforming growth factor (TGF)-β signaling in cardiac remodeling. J. Mol. Cell. Cardiol. 51 (4), 600–606. 10.1016/j.yjmcc.2010.10.033 21059352 PMC3072437

[B11] Fatkin D. MacRae C. Sasaki T. Wolff M. R. Porcu M. Frenneaux M. (1999). Missense mutations in the rod domain of the lamin A/C gene as causes of dilated cardiomyopathy and conduction-system disease. N. Engl. J. Med. 341 (23), 1715–1724. 10.1056/nejm199912023412302 10580070

[B12] Fitzgerald M. Saville B. R. Lewis R. J. (2015). Decision curve analysis. Jama 313 (4), 409–410. 10.1001/jama.2015.37 25626037

[B13] Gaertner A. Schwientek P. Ellinghaus P. Summer H. Golz S. Kassner A. (2012). Myocardial transcriptome analysis of human arrhythmogenic right ventricular cardiomyopathy. Physiol. Genomics 44 (1), 99–109. 10.1152/physiolgenomics.00094.2011 22085907

[B14] Gerull B. Gramlich M. Atherton J. McNabb M. Trombitás K. Sasse-Klaassen S. (2002). Mutations of TTN, encoding the giant muscle filament titin, cause familial dilated cardiomyopathy. Nat. Genet. 30 (2), 201–204. 10.1038/ng815 11788824

[B15] Goetze J. P. Bruneau B. G. Ramos H. R. Ogawa T. de Bold M. K. de Bold A. J. (2020). Cardiac natriuretic peptides. Nat. Rev. Cardiol. 17 (11), 698–717. 10.1038/s41569-020-0381-0 32444692

[B16] Guo W. Feng W. Fan X. Huang J. Ou C. Chen M. (2021). Osteomodulin is a potential genetic target for hypertrophic cardiomyopathy. Biochem. Genet. 59 (5), 1185–1202. 10.1007/s10528-021-10050-1 33715137

[B17] Han L. Yuan Y. Zheng S. Yang Y. Li J. Edgerton M. E. (2014). The Pan-Cancer analysis of pseudogene expression reveals biologically and clinically relevant tumour subtypes. Nat. Commun. 5, 3963. 10.1038/ncomms4963 24999802 PMC4339277

[B18] Iwata Y. Ito S. Wakabayashi S. Kitakaze M. (2020). TRPV2 channel as a possible drug target for the treatment of heart failure. Lab. Invest. 100 (2), 207–217. 10.1038/s41374-019-0349-z 31857697

[B19] Japp A. G. Gulati A. Cook S. A. Cowie M. R. Prasad S. K. (2016). The diagnosis and evaluation of dilated cardiomyopathy. J. Am. Coll. Cardiol. 67 (25), 2996–3010. 10.1016/j.jacc.2016.03.590 27339497

[B20] Johnson J. D. Fugman D. A. (1983). Calcium and calmodulin antagonists binding to calmodulin and relaxation of coronary segments. J. Pharmacol. Exp. Ther. 226 (2), 330–334. 6875846

[B21] Karsoliya S. (2012). Approximating number of hidden layer neurons in multiple hidden layer BPNN architecture. Int. J. Eng. Trends Technol. 3 (6), 714–717.

[B22] Kaundal R. Kapoor A. S. Raghava G. P. (2006). Machine learning techniques in disease forecasting: A case study on rice blast prediction. BMC Bioinforma. 7, 485. 10.1186/1471-2105-7-485 PMC1647291 17083731

[B23] Kuleshov M. V. Jones M. R. Rouillard A. D. Fernandez N. F. Duan Q. Wang Z. (2016). Enrichr: A comprehensive gene set enrichment analysis web server 2016 update. Nucleic Acids Res. 44 (W1), W90–W97. 10.1093/nar/gkw377 27141961 PMC4987924

[B24] Langfelder P. Horvath S. (2008). Wgcna: an R package for weighted correlation network analysis. BMC Bioinforma. 9, 559. 10.1186/1471-2105-9-559 PMC2631488 19114008

[B25] Luo W. Pant G. Bhavnasi Y. K. Blanchard S. G. Jr. Brouwer C. (2017). Pathview web: User friendly pathway visualization and data integration. Nucleic Acids Res. 45 (W1), W501–w508. 10.1093/nar/gkx372 28482075 PMC5570256

[B26] MacCarthy E. P. Bloomfield S. S. (1983). Labetalol: A review of its pharmacology, pharmacokinetics, clinical uses and adverse effects. Pharmacotherapy 3 (4), 193–219. 10.1002/j.1875-9114.1983.tb03252.x 6310529

[B27] Mahmaljy H. Yelamanchili V. S. Singhal M. (2022). “Dilated cardiomyopathy,” in *StatPearls*. (Treasure island (FL) (Florida: StatPearls Publishing). 28722940

[B28] Mazzarotto F. Tayal U. Buchan R. J. Midwinter W. Wilk A. Whiffin N. (2020). Reevaluating the genetic contribution of monogenic dilated cardiomyopathy. Circulation 141 (5), 387–398. 10.1161/circulationaha.119.037661 31983221 PMC7004454

[B29] McDonagh T. A. Metra M. Adamo M. Gardner R. S. Baumbach A. Böhm M. (2022). 2021 ESC Guidelines for the diagnosis and treatment of acute and chronic heart failure: Developed by the Task Force for the diagnosis and treatment of acute and chronic heart failure of the European Society of Cardiology (ESC). With the special contribution of the Heart Failure Association (HFA) of the ESC. Eur. J. Heart Fail. 24 (1), 4–131. 10.1002/ejhf.2333 35083827

[B30] McNally E. M. Mestroni L. (2017). Dilated cardiomyopathy: Genetic determinants and mechanisms. Circ. Res. 121 (7), 731–748. 10.1161/circresaha.116.309396 28912180 PMC5626020

[B31] Merlo M. Pivetta A. Pinamonti B. Stolfo D. Zecchin M. Barbati G. (2014). Long-term prognostic impact of therapeutic strategies in patients with idiopathic dilated cardiomyopathy: Changing mortality over the last 30 years. Eur. J. Heart Fail. 16 (3), 317–324. 10.1002/ejhf.16 24464640

[B32] Ngo D. Benson M. D. Long J. Z. Chen Z. Z. Wang R. Nath A. K. (2021). Proteomic profiling reveals biomarkers and pathways in type 2 diabetes risk. JCI Insight 6 (5), e144392. 10.1172/jci.insight.144392 33591955 PMC8021115

[B33] Nomura S. Satoh M. Fujita T. Higo T. Sumida T. Ko T. (2018). Cardiomyocyte gene programs encoding morphological and functional signatures in cardiac hypertrophy and failure. Nat. Commun. 9 (1), 4435. 10.1038/s41467-018-06639-7 30375404 PMC6207673

[B34] Olson R. S. Cava W. Mustahsan Z. Varik A. Moore J. H. (2018). Data-driven advice for applying machine learning to bioinformatics problems. Pac. Symp. Biocomput. 23, 192–203. 29218881 PMC5890912

[B35] Pan X. Jin X. Wang J. Hu Q. Dai B. (2021). Placenta inflammation is closely associated with gestational diabetes mellitus. Am. J. Transl. Res. 13 (5), 4068–4079. 34149999 PMC8205654

[B36] Ruan F. Ding X. Li H. Wang Y. Ye K. Kan H. (2021). Back propagation neural network model for medical expenses in patients with breast cancer. Math. Biosci. Eng. 18 (4), 3690–3698. 10.3934/mbe.2021185 34198407

[B37] Sato N. Tamada Y. Yu G. Okuno Y. (2022). CBNplot: Bayesian network plots for enrichment analysis. Bioinformatics 38 (10), 2959–2960. 10.1093/bioinformatics/btac175 35561164 PMC9113354

[B38] Schultheiss H. P. Fairweather D. Caforio A. L. P. Escher F. Hershberger R. E. Lipshultz S. E. (2019). Dilated cardiomyopathy. Nat. Rev. Dis. Prim. 5 (1), 32. 10.1038/s41572-019-0084-1 31073128 PMC7096917

[B39] Schwientek P. Ellinghaus P. Steppan S. D'Urso D. Seewald M. Kassner A. (2010). Global gene expression analysis in nonfailing and failing myocardium pre- and postpulsatile and nonpulsatile ventricular assist device support. Physiol. Genomics 42 (3), 397–405. 10.1152/physiolgenomics.00030.2010 20460602

[B40] Schwinger R. H. Böhm M. Erdmann E. (1992). Inotropic and lusitropic dysfunction in myocardium from patients with dilated cardiomyopathy. Am. Heart J. 123 (1), 116–128. 10.1016/0002-8703(92)90755-k 1309621

[B41] Seferović P. M. Polovina M. Bauersachs J. Arad M. Ben Gal T. Lund L. H. (2019). Heart failure in cardiomyopathies: A position paper from the heart failure association of the European society of cardiology. Eur. J. Heart Fail. 21 (5), 553–576. 10.1002/ejhf.1461 30989768

[B42] Seidman J. G. Seidman C. (2001). The genetic basis for cardiomyopathy: From mutation identification to mechanistic paradigms. Cell. 104 (4), 557–567. 10.1016/s0092-8674(01)00242-2 11239412

[B43] Sheridan R. P. Wang W. M. Liaw A. Ma J. Gifford E. M. (2016). Extreme gradient boosting as a method for quantitative structure-activity relationships. J. Chem. Inf. Model. 56 (12), 2353–2360. 10.1021/acs.jcim.6b00591 27958738

[B44] Skenteris N. T. Seime T. Witasp A. Karlöf E. Wasilewski G. B. Heuschkel M. A. (2022). Osteomodulin attenuates smooth muscle cell osteogenic transition in vascular calcification. Clin. Transl. Med. 12 (2), e682. 10.1002/ctm2.682 35184400 PMC8858609

[B45] Swedberg K. Kjekshus J. Group C. T. S. (1987). Effects of enalapril on mortality in severe congestive heart failure. Results of the cooperative north scandinavian enalapril survival study (CONSENSUS). N. Engl. J. Med. 316 (23), 1429–1435. 10.1056/nejm198706043162301 2883575

[B46] Thompson S. A. Arden S. A. Marshall G. Wingrove P. B. Whiting P. J. Wafford K. A. (1999). Residues in transmembrane domains I and II determine gamma-aminobutyric acid type AA receptor subtype-selective antagonism by furosemide. Mol. Pharmacol. 55 (6), 993–999. 10.1124/mol.55.6.993 10347239

[B47] Tian Y. Yang J. Lan M. Zou T. (2020). Construction and analysis of a joint diagnosis model of random forest and artificial neural network for heart failure. Aging (Albany NY) 12 (24), 26221–26235. 10.18632/aging.202405 33401250 PMC7803554

[B48] Weintraub R. G. Semsarian C. Macdonald P. (2017). Dilated cardiomyopathy. Lancet 390 (10092), 400–414. 10.1016/s0140-6736(16)31713-5 28190577

[B50] Yang L. Wu H. Jin X. Zheng P. Hu S. Xu X. (2020). Study of cardiovascular disease prediction model based on random forest in eastern China. Sci. Rep. 10 (1), 5245. 10.1038/s41598-020-62133-5 32251324 PMC7090086

[B51] Zhang X. Lan Y. Xu J. Quan F. Zhao E. Deng C. (2019). CellMarker: A manually curated resource of cell markers in human and mouse. Nucleic Acids Res. 47 (D1), D721–d728. 10.1093/nar/gky900 30289549 PMC6323899

[B52] Zhang Y. Fu C. Zhao S. Jiang H. Li W. Liu X. (2022). PRELP promotes myocardial fibrosis and ventricular remodelling after acute myocardial infarction by the wnt/β-catenin signalling pathway. Cardiovasc. J. Afr. 33, 228–233. 10.5830/cvja-2022-001 35788244 PMC9887443

[B53] Zheng Y. Liu Z. Yang X. Weng S. Xu H. Guo C. (2022). Exploring key genes to construct a diagnosis model of dilated cardiomyopathy. Front. Cardiovasc. Med. 9, 865096. 10.3389/fcvm.2022.865096 35571180 PMC9091505

