# Supplementary figures and images for: Integrated analysis of WGCNA and machine learning identified diagnostic biomarkers in dilated cardiomyopathy with heart failure
Source: Front Cell Dev Biol. 2022 Dec 5;10:1089915. doi: 10.3389/fcell.2022.1089915 (PMC9760806; doi:10.3389/fcell.2022.1089915)

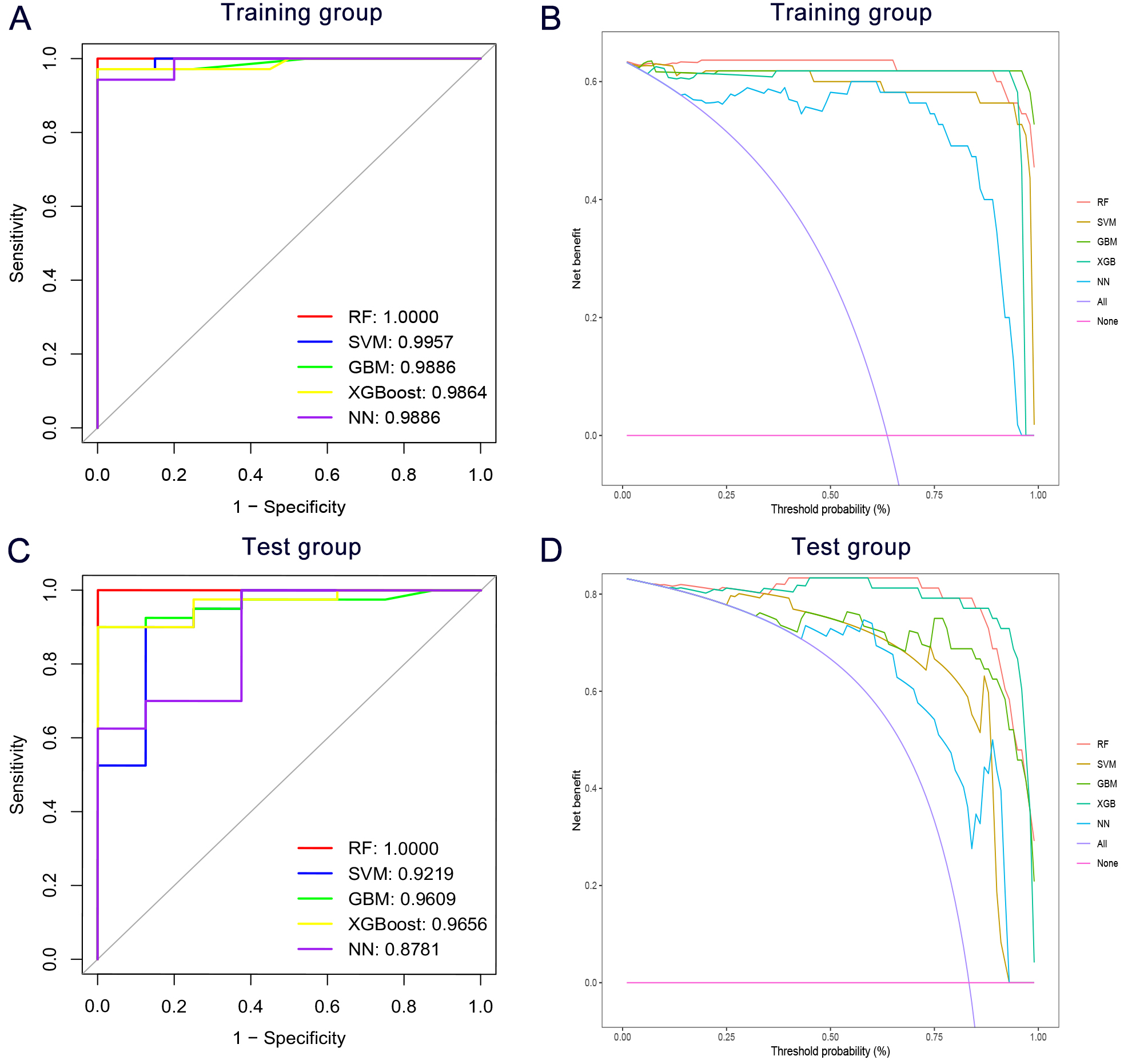

Supplement: Supplementary file 1 [file Image3.JPEG]

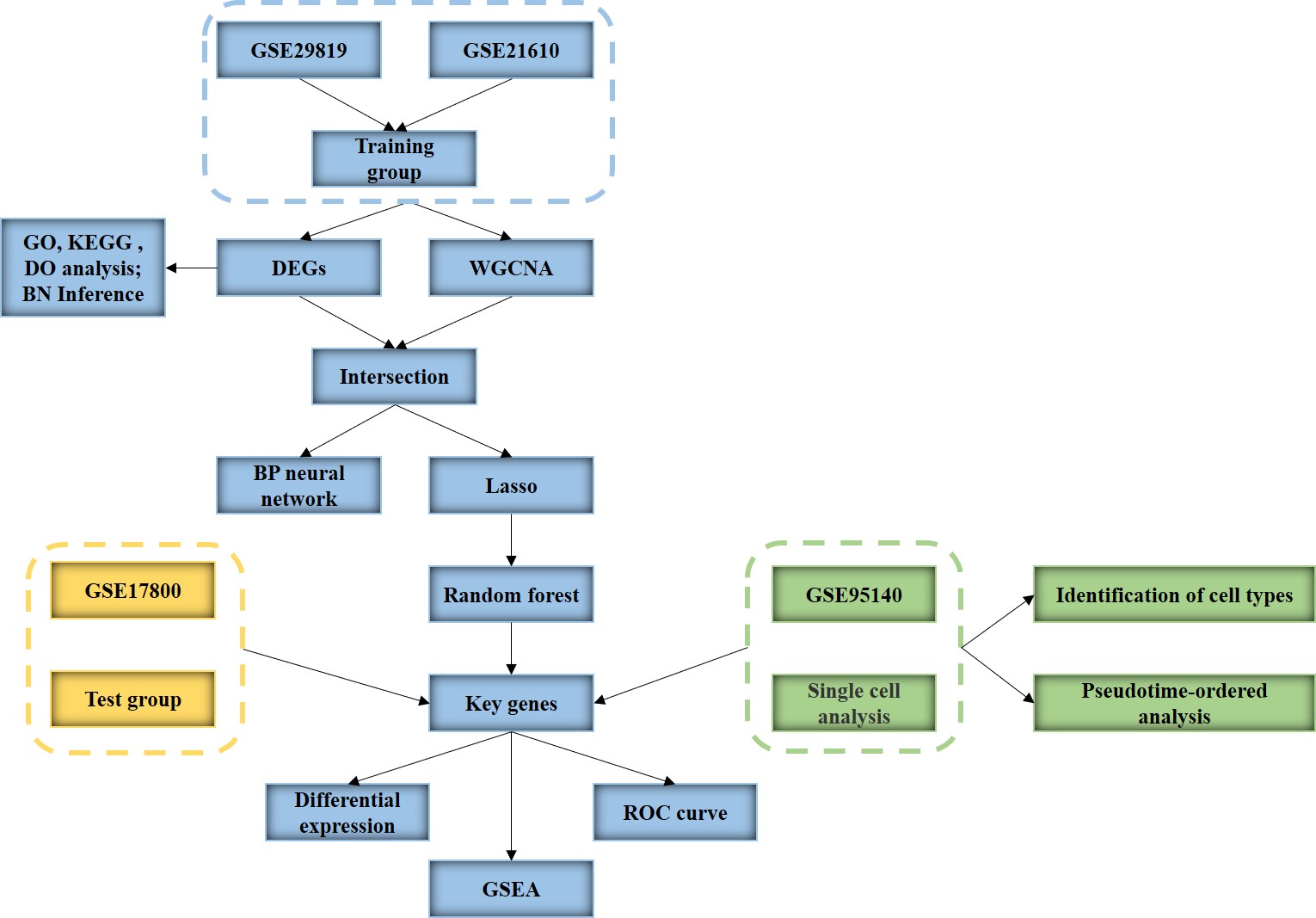

Supplement: Supplementary file 2 [file Image1.JPEG]

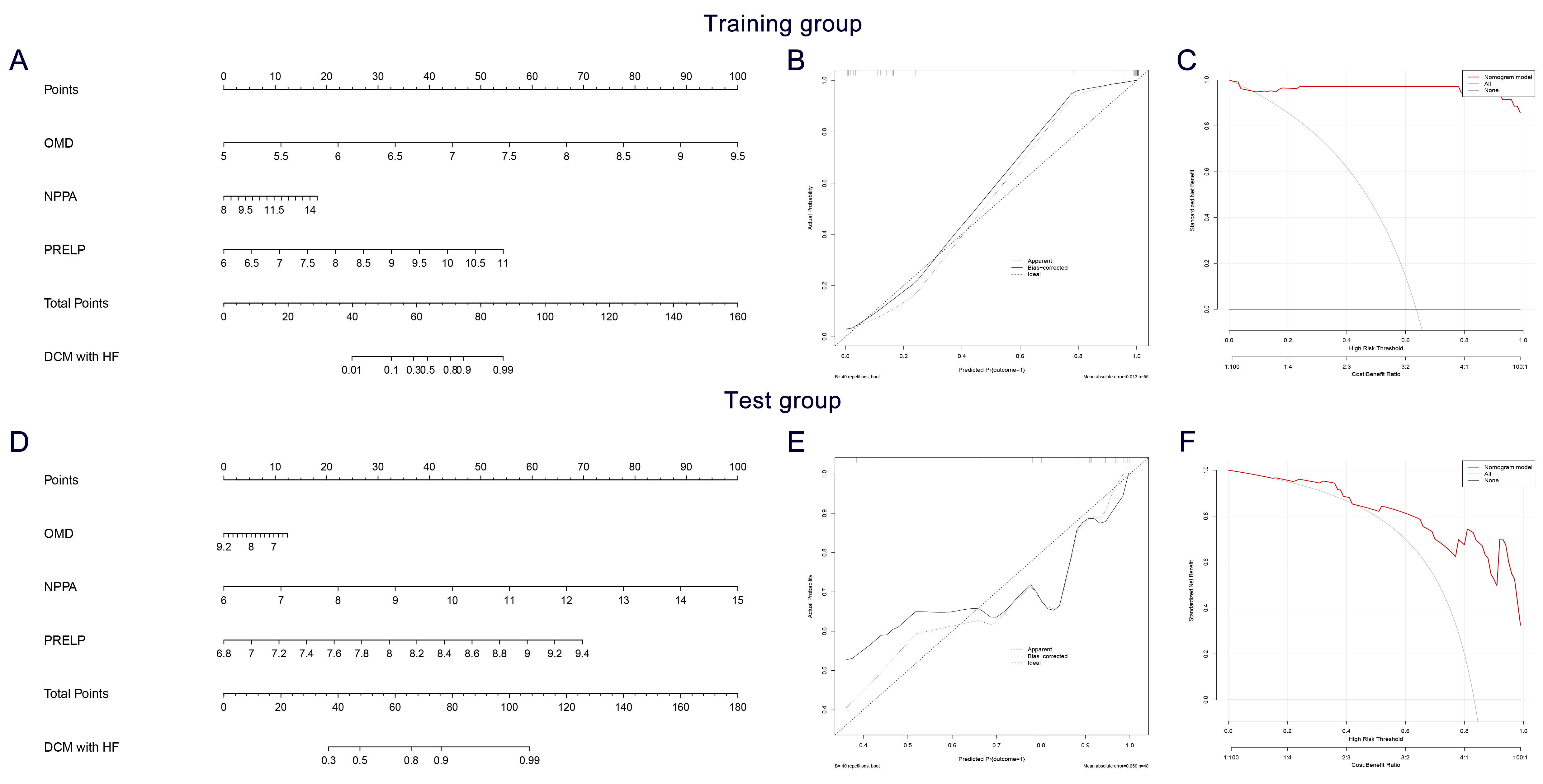

Supplement: Supplementary file 3 [file Image4.JPEG]

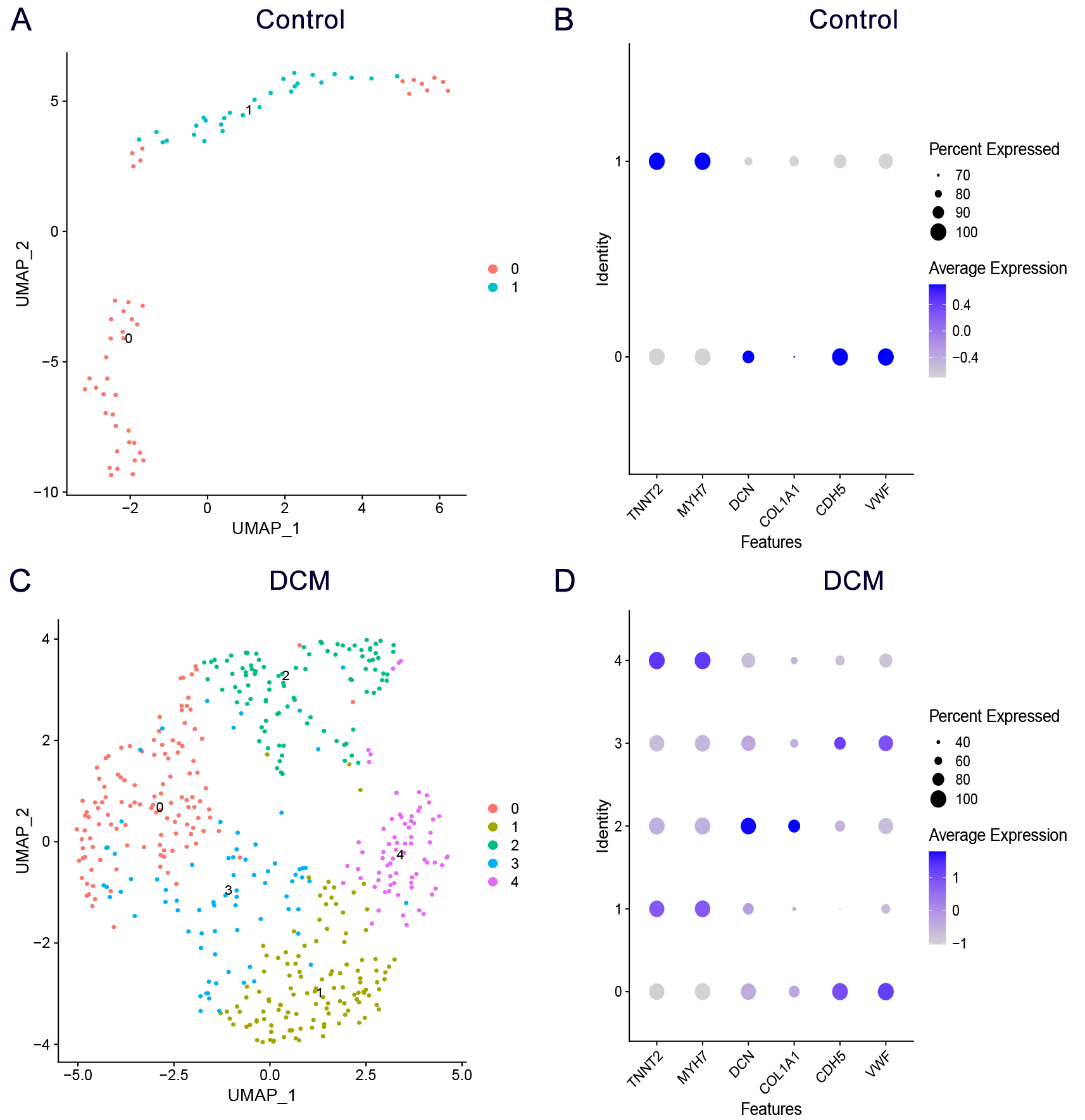

Supplement: Supplementary file 4 [file Image7.JPEG]

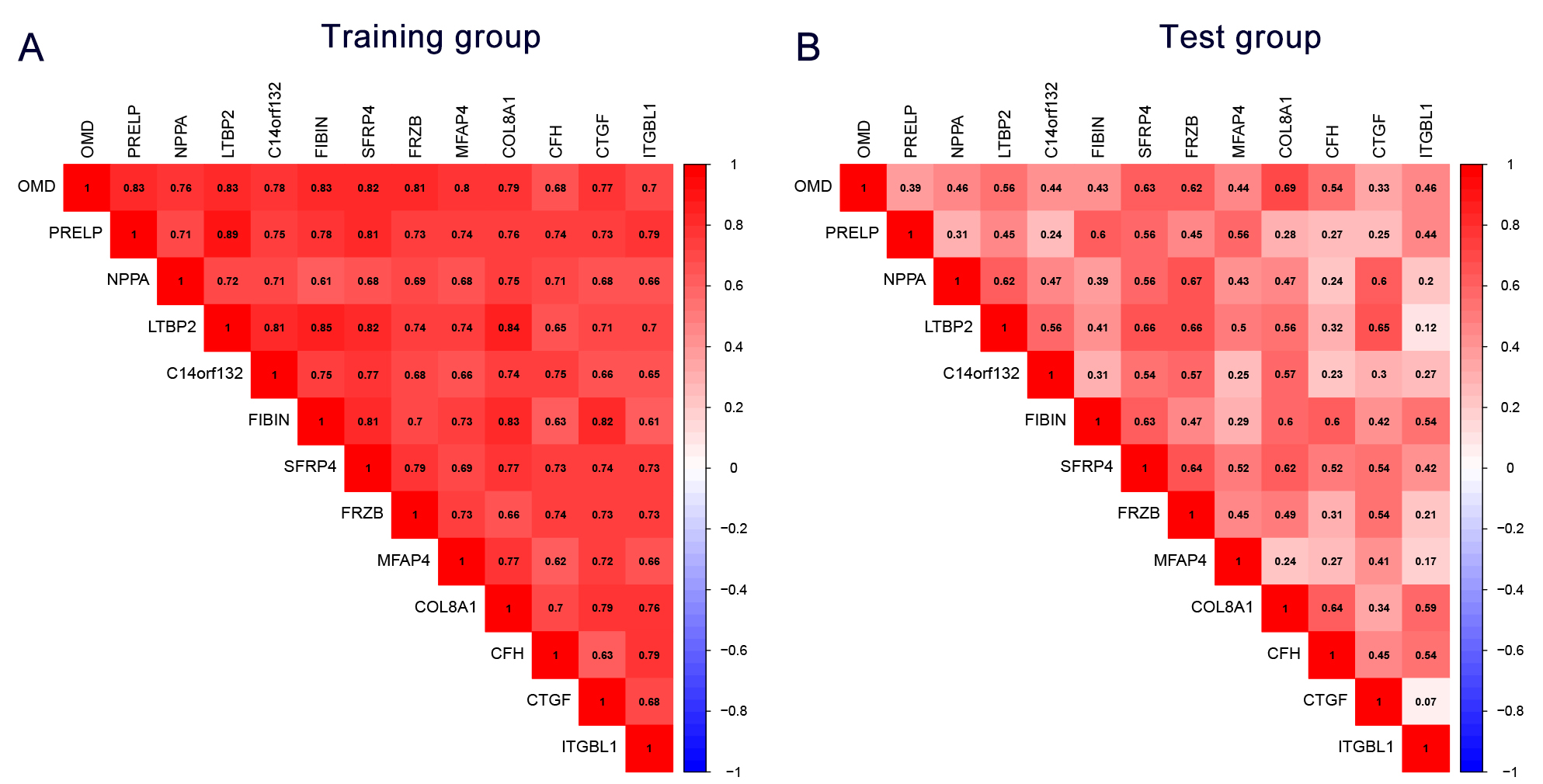

Supplement: Supplementary file 5 [file Image2.JPEG]

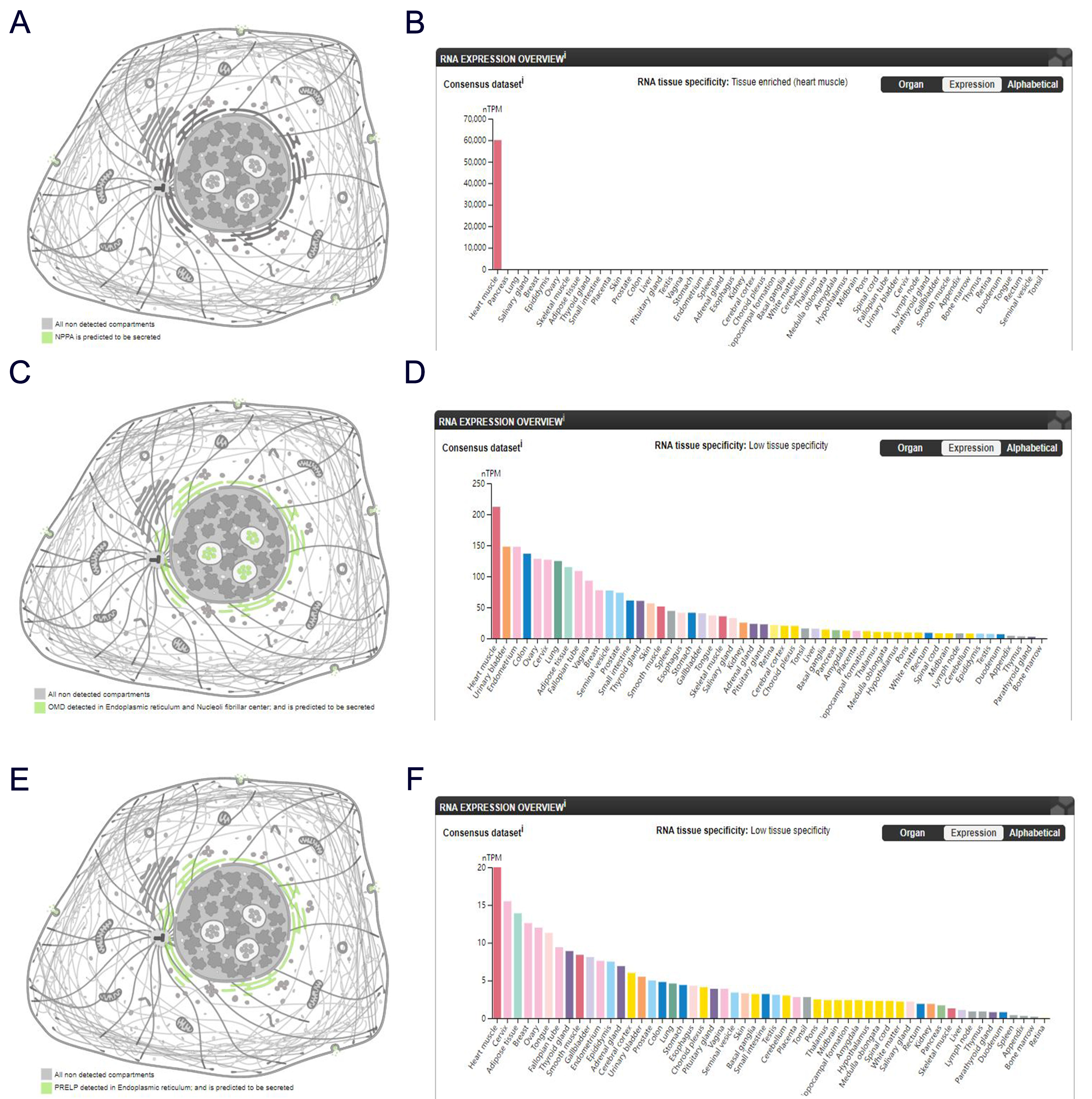

Supplement: Supplementary file 6 [file Image5.JPEG]

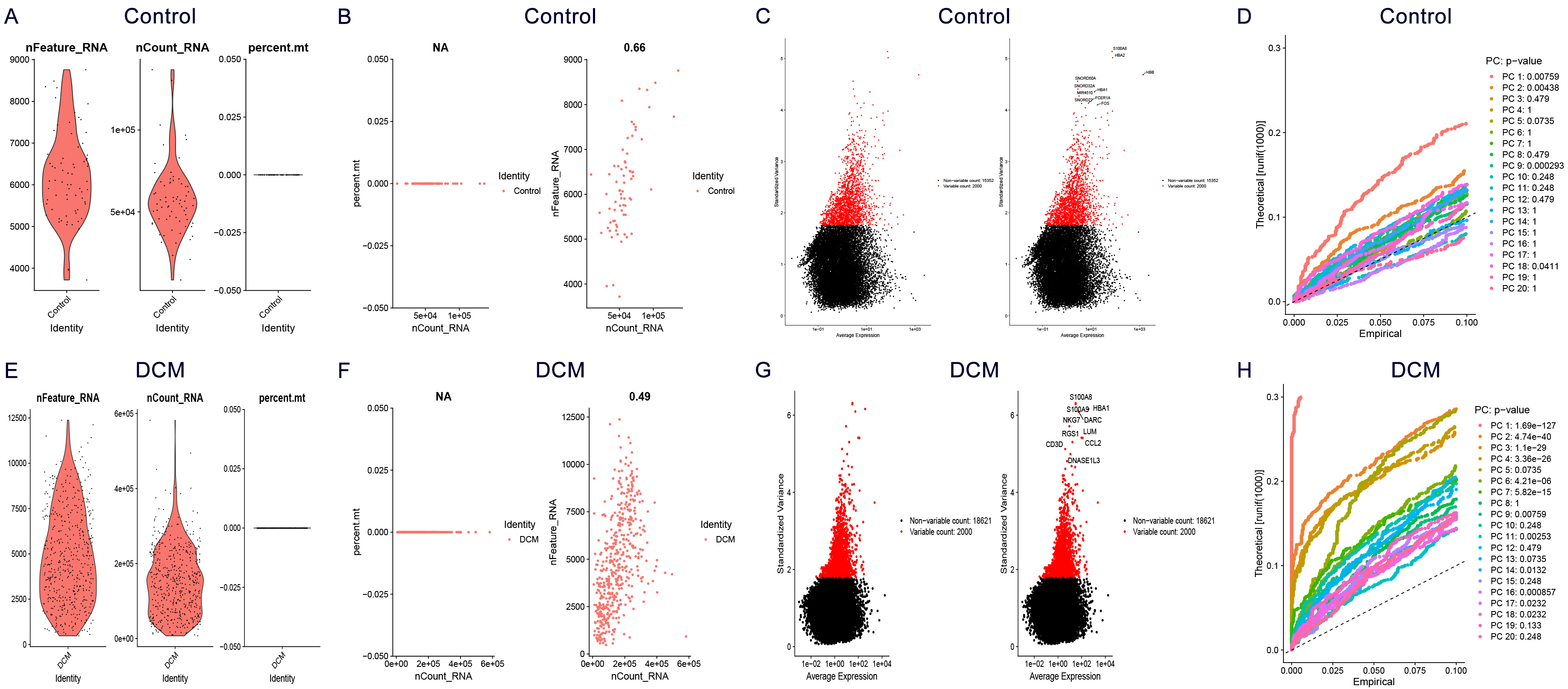

Supplement: Supplementary file 8 [file Image6.JPEG]
