# Supplementary material for: Integrated analysis of WGCNA and machine learning identified diagnostic biomarkers in dilated cardiomyopathy with heart failure
Source: Front Cell Dev Biol. 2022 Dec 5;10:1089915. doi: 10.3389/fcell.2022.1089915 (PMC9760806; doi:10.3389/fcell.2022.1089915)
Supplement: Supplementary file 7 [file DataSheet1.DOCX]

**SUPPLEMENT TABLE 1**. Brier score for measuring calibration of five classifiers in training and test groups;

| Classifiers | Brier score in training group | Brier score in test group |
| --- | --- | --- |
| RF | 0.004514 | 0.016781 |
| SVM | 0.024914 | 0.068804 |
| GBM | 0.017429 | 0.065676 |
| XGB | 0.021269 | 0.020802 |
| NN | 0.061401 | 0.095606 |

**SUPPLEMENT TABLE 2**. Top 10 Predictive drugs significantly associated with NPPA;

| Index | | Drug name | Adjusted *P* value | Odds Ration | Combined Score |
| --- | --- | --- | --- | --- | --- |
| 1 | cyclic gmp CTD 00006063 | | 0.01948 | 832.71 | 5196.55 |
| 2 | glycerol CTD 00006038 | | 0.01948 | 832.71 | 5196.55 |
| 3 | alprostadil CTD 00005360 | | 0.01948 | 666.07 | 4018.42 |
| 4 | labetalol CTD 00006196 | | 0.01948 | 624.41 | 3729.25 |
| 5 | felodipine CTD 00007084 | | 0.01948 | 525.74 | 3054.59 |
| 6 | irbesartan CTD 00002968 | | 0.01948 | 525.74 | 3054.59 |
| 7 | furosemide CTD 00006012 | | 0.01948 | 453.98 | 2574.28 |
| 8 | Bonuten CTD 00005895 | | 0.01948 | 416.1 | 2324.86 |
| 9 | naloxone CTD 00006373 | | 0.01948 | 399.44 | 2216.11 |
| 10 | Epirubicin CTD 00007057 | | 0.01955 | 356.59 | 1939.49 |

**SUPPLEMENT TABLE 3**. Gene list of TGF-β signaling pathway;

| KEGG pathway name | Gene list |
| --- | --- |
| TGF-β signaling pathway | CHRD, NOG, NBL1, MICOS10-NBL1, GREM1, GREM2, THBS1, DCN, FMOD, LEFTY1, LEFTY2, FST, BMP2, BMP4, BMP6, INHBB, BMP5, BMP7, BMP8B, BMP8, GDF5, GDF6, GDF7, AMH, THSD4, FBN1, LTBP1, TGFB1, TGFB2, TGFB3, INHBA, INHBC, INHBE, NODAL, NEO1, HJV, BMPR1A, BMPR1B, ACVR1, BMPR2, ACVR2A, RGMA, RGMB, AMHR2, TGFBR1, TGFBR2, ACVR1B, ACVR2B, ACVR1C, BAMBI, SMAD1, SMAD5, SMAD9, SMAD2, SMAD3, SMAD4, SMAD6, SMAD7, SMURF1, SMURF2, ZFYVE9, ZFYVE16, HAMP, ID1, ID2, ID3, ID4, RBL1, E2F4, E2F5, TFDP1, CREBBP, EP300, SP1, TGIF1, TGIF2, MYC, CDKN2B, PITX2, RBX1, CUL1, SKP1, MAPK1, MAPK3, IFNG, TNF, RHOA, ROCK1, PPP2R1B, PPP2R1A, PPP2CA, PPP2CB, RPS6KB1, RPS6KB2 |

**SUPPLEMENT TABLE 4**. TGF-β signaling pathway-related genes significantly correlated with three key genes;

| Key genes | The significant correlation genes in TGF-β signaling pathway (\|*R*\| > 0.3, *P* < 0.05) |
| --- | --- |
| NPPA | INHBA, ID4, SMURF1, TGFB2, FBN1, TGFBR1, TGFB3, SMAD7, SMAD9, SMAD2, THBS1, NBL1, NOG, SMURF2, E2F4, BMP7 |
| OMD | DCN, FMOD, SMAD9, ID4, INHBA, FST, BMP2, TGFB3, TGFBR1, SMAD7, TFDP1, BMP4, ACVR1C, SMURF2, GREM2, BMP6, RHOA, HAMP, PPP2R1A, BMP8B |
| PRELP | FMOD, TGFB3, DCN, LTBP1, FST, BMP2, INHBB, SMAD7, NBL1, SMURF1, CREBBP, SMAD9, SP1, ID4, BMP4, BAMBI, FBN1, TGFBR2, CHRD, GREM2, NOG, INHBA, RHOA, NEO1, TGFB1, BMP8B |
